# Breakdown in membrane asymmetry regulation leads to monocyte recognition of *P*. *falciparum*-infected red blood cells

**DOI:** 10.1371/journal.ppat.1009259

**Published:** 2021-02-18

**Authors:** Merryn Fraser, Weidong Jing, Stefan Bröer, Florian Kurth, Leif-Erik Sander, Kai Matuschewski, Alexander G. Maier

**Affiliations:** 1 Research School of Biology, The Australian National University, Canberra, Australia; 2 Department of Molecular Parasitology, Institute of Biology, Humboldt University, Berlin, Germany; 3 Department of Infectious Diseases and Pulmonary Medicine, Charité-Universitätsmedizin Berlin, Germany; 4 Department of Tropical Medicine, Bernhard Nocht Institute for Tropical Medicine, Hamburg, Germany; 5 Department of Medicine, University Medical Centre Hamburg-Eppendorf, Hamburg, Germany; Johns Hopkins University Bloomberg School of Public Health, UNITED STATES

## Abstract

The human malaria parasite *Plasmodium falciparum* relies on lipids to survive; this makes its lipid metabolism an attractive drug target. The lipid phosphatidylserine (PS) is usually confined to the inner leaflet of the red blood cell membrane (RBC) bilayer; however, some studies suggest that infection with the intracellular parasite results in the presence of this lipid in the RBC membrane outer leaflet, where it could act as a recognition signal to phagocytes. Here, we used fluorescent lipid analogues and probes to investigate the enzymatic reactions responsible for maintaining asymmetry between membrane leaflets, and found that in parasitised RBCs the maintenance of membrane asymmetry was partly disrupted, and PS was increased in the outer leaflet. We examined the underlying causes for the differences between uninfected and infected RBCs using fluorescent dyes and probes, and found that calcium levels increased in the infected RBC cytoplasm, whereas membrane cholesterol was depleted from the erythrocyte plasma membrane. We explored the resulting effect of PS exposure on enhanced phagocytosis by monocytes, and show that infected RBCs must expend energy to limit phagocyte recognition, and provide experimental evidence that PS exposure contributes to phagocytic recognition of *P*. *falciparum*-infected RBCs. Together, these findings underscore the pivotal role for PS exposure on the surface of *Plasmodium falciparum*-infected erythrocytes for *in vivo* interactions with the host immune system, and provide a rationale for targeted antimalarial drug design.

## Author summary

Malaria remains the deadliest parasitic disease in the world despite years of sustained effort, new drug development, and a greater understanding of the parasite and its interactions with its host. We examined a series of interconnected processes, from lipid and calcium sequestration through to recognition by immune cells. We show here that the uptake of cholesterol and calcium ions induce changes to the parasitised host red blood cell. These changes affect the activity of lipid-transporting enzymes in the host cell, which keep certain phospholipids on specific sides of the membrane: a concept called membrane asymmetry. We show that the parasite causes activation of a calcium- and cholesterol-sensitive enzyme, scramblase, so phospholipids are scrambled back and forth. Therefore, the lipid phosphatidylserine becomes exposed in the membrane outer layer, acting as an ‘eat me’ signal to phagocytes including monocytes. Throughout our study, we show that the infected cell is expending energy to compensate for the processes which ultimately lead to phagocytosis. But despite these compensatory processes, a portion of parasites are ingested by monocytes. Our systematic exploration of this pathway addresses contradictory findings from past reports, and exposes vulnerabilities in the parasite’s metabolism that could be used for targeted drug design.

## Introduction

Malaria is a devastating infectious disease, caused by blood infection with *Plasmodium* parasites [1,2]. During the asexual phase of their lifecycle, *Plasmodium* parasites invade and develop inside red blood cells (RBCs). The parasites induce changes to their host cells, which can be broadly understood as changes that assist them to acquire nutrients and to avoid the host immune system, which together contribute to the parasite’s virulence [3,4]. The high mortality and morbidity of *Plasmodium falciparum*, the species responsible for the vast majority of malaria-related deaths, is in part due to the extent of the changes this species induces. In particular, the parasite exports proteins which allow the host RBC to cytoadhere to the vascular endothelium. This prevents the infected RBC from circulating through the spleen, where it would otherwise be vulnerable to immune clearance [5,6]. The parasite also induces changes to the permeability of its host cell to enhance the uptake of nutrients, though this may leave the parasite vulnerable to other consequences of altered solute permeability [7].

Healthy eukaryotic cells, including RBCs, maintain an asymmetry in phospholipid distribution between the inner and outer leaflets of the membrane bilayer [8,9]. The phospholipid asymmetry gives the inner and outer membrane leaflets different biophysical properties, providing the basis for a variety of cellular characteristics and processes such as membrane curvature, differences in charge, and vesicle budding and fusion [10–13]. The aminophospholipids phosphatidylserine (PS) and phosphatidylethanolamine (PE) are almost exclusively present in the inner (cytoplasmic) leaflet, whereas phosphatidylcholine (PC) is predominantly present in the outer (exofacial) leaflet. The breakdown of this asymmetry can have dramatic consequences for the cell. For example, the presence of PS in the outer leaflet (‘PS exposure’) is a hallmark of apoptosis in a variety of cell types, and acts as a signal for monocyte and macrophage recognition and binding, resulting in cell destruction [14–17]. PS exposure is also a key mechanism in platelet activation, which initiates coagulation cascades [12,18].

Membrane asymmetry is established and regulated by three classes of enzymes: flippase, floppase, and scramblase [19]. Flippase enzymes, also called aminophospholipid translocases, are responsible for transporting (‘flipping’) PS (and to a lesser extent PE) from the outer leaflet to the inner leaflet. Flippase proteins have putatively been identified as P4-type ATPases [20–22]; three members of this enzyme family (ATP11A, ATP11B, ATP11C) are present in human RBCs [23–25]. These proteins utilise PS and PE, but not PC, as substrates [20,21,23]. Floppase enzymes function in the opposite direction, externalising PC from the inner leaflet to the outer leaflet; ABC transporters have been implicated in this function [26,27]. Both of these classes of enzymes require the hydrolysis of ATP, and are generally active throughout the life of a cell. In contrast, scramblase enzymes can translocate lipids in either direction, regardless of the head group. Scramblases do not depend on ATP hydrolysis to function, and they can rapidly equilibrate lipids across the membrane, leading to a collapse of phospholipid asymmetry [28–30]. Scramblase activity is repressed in healthy cells, and is activated when intracellular Ca^2+^ concentrations increase, or by caspase-mediated cleavage. The identity of proteins responsible for scramblase activity in human RBCs is still under debate [30–33].

PS exposure in RBCs infected with *Plasmodium* parasites (iRBCs) has been extensively examined, but studies have reached different conclusions (for example, [34–45]). More recent studies tend to conclude that iRBCs have a higher level of PS exposure than their uninfected counterparts (uRBCs), with increasing levels of exposure as the parasite develops [46–49], although these later studies did not include glucose in their assay buffers, which may artificially increase the level of PS exposure [40,50].

PS exposure can be induced by changes to cytoplasmic Ca^2+^. Healthy RBCs normally maintain a very low intracellular concentration of free Ca^2+^ (30–60 nM), despite a high extracellular concentration in blood plasma (1.8 mM) [51]. This steep concentration gradient is due to the low Ca^2+^ permeability of the RBC membrane, and the action of a membrane pump which extrudes Ca^2+^ [52]. However, *P*. *falciparum* parasites rely on the uptake of extracellular calcium and maintenance of intracellular stores to invade, develop, and egress from the host RBC [53–56]. Therefore, infection with *P*. *falciparum* results in an increase in intracellular Ca^2+^ in iRBCs [57–60]. Most studies to date have focused on calcium regulation in the parasite itself, rather than exploring the calcium concentration in the host RBC cytoplasm (for example, [61–63]). The few studies which explicitly looked at calcium in the cytoplasm of iRBCs have found contradictory results, with Rohrbach *et al*. [59] finding that calcium was lower in the iRBC cytoplasm than uRBCs, and Zipprer *et al*. [58] finding that it was higher.

Given the importance of phospholipid asymmetry in RBCs, and the long-standing controversy and contradiction in previous studies on PS exposure and Ca^2+^ regulation in iRBCs, we set out a systematic analysis of phospholipid uptake and membrane distribution during infection and asexual replication of *Plasmodium falciparum* within host erythrocytes. We found that the changes the parasite induces to the host RBC result in increased scramblase activity, leading to PS exposure and ultimately to phagocytic clearance of iRBCs.

## Results

### Infected RBCs internalise more fluorescent phospholipids than uninfected RBCs

To investigate lipid translocation in iRBCs, we performed lipid internalisation assays, using analogues of PS, PE, and PC conjugated to the fluorophore nitrobenzoxadiazole (NBD; **S1A Fig**) [64].

In this assay, NBD-PS, NBD-PE, or NBD-PC was added to the extracellular environment, where the analogues passively incorporate into the RBC membrane outer leaflet. After a 20-minute incubation, NBD-lipids remaining in the outer leaflet were extracted with lipid-free bovine serum albumin (BSA). The fluorescence intensity after BSA extraction represents the fluorescently labelled lipids which have flipped to the inner leaflet, hence reflecting the combined activity of RBC scramblase and flippase proteins.

**Fig 1:**
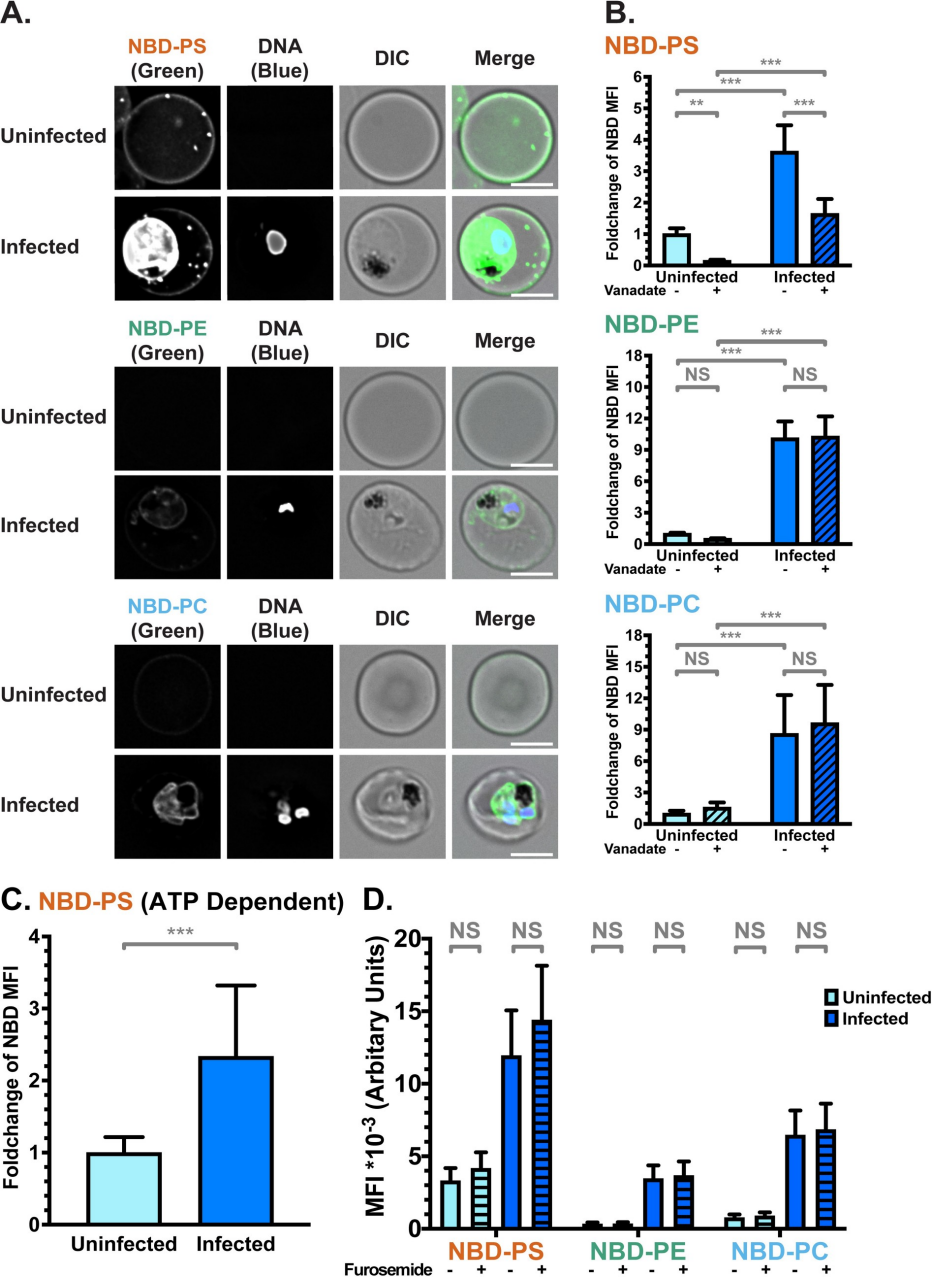
NBD-lipid internalisation at the red blood cell (RBC) membrane differs between uninfected (uRBCs) and infected (iRBCs) cells. (**A**) Subcellular localisation of NBD-lipids after internalisation, visualised by deconvolution fluorescence microscopy. NBD-lipid fluorescence was detected at 475 nm (ex)/ 525 nm (em) and Hoechst fluorescence (parasite DNA) was detected at 390 nm (ex)/ 435 nm (em). Shown are representative images for uninfected (top) and infected (bottom) RBCs incubated with NBD-PS (upper rows), NBD-PE (centre rows) and NBD-PC (lower rows) after extraction of NBD-lipids remaining in the outer layer. Scale bar = 4 μm. (**B**) NBD-lipid internalisation, measured by fold-change of NBD mean fluorescence intensity (MFI) of whole cells in flow cytometry after extraction of NBD-lipids remaining in the outer layer. Cells were treated with 0.5 mM vanadate in calcium-free media to measure only the ATP-independent portion of internalisation. Shown are percentage of mean values (± S.D.) NS = not significant; ** = p < 0.01; *** = p < 0.001 (ANOVA). *n* = 3 independent experiments. (**C**) ATP-dependent fraction of NBD-PS internalisation in RBCs, calculated from the difference between total (untreated) and ATP-independent (vanadate treated) fractions (see Methods). Shown is normalised mean fluorescence intensity (± S.D.), *** = p < 0.001 (Mann-Whitney Test). *n* = 3 independent experiments. (**D**) NBD-lipid internalisation with treatment of 100 μM furosemide, measured by mean fluorescence intensity (MFI). Units are arbitrary. Shown are Least Square Means (± 95% Confidence Interval). NS = not significant (Difference of Least Square Means test). *n* = 3 independent experiments.

Using deconvolution fluorescence microscopy, we noticed strong fluorescent signals for all three lipid classes in iRBCs infected with *P*. *falciparum* 3D7 wildtype parasite strain, distinguished from uRBCs by fluorescence from the nucleic acid stain, Hoechst 33342. Regardless of which NBD-lipid was used, the weak fluorescence of uRBCs was concentrated in the RBC plasma membrane (**Fig 1A**). The plasma membrane of iRBC also displayed a fluorescent signal for all three lipids, and in addition, fluorescent lipids also localised in or around the intracellular parasite (**Figs 1A** and **S2**).

In order to quantify and compare the movement of NBD-lipids to the internal layer of the membrane, we measured mean fluorescence intensity (MFI) of whole cells using flow cytometry, distinguishing uRBCs and iRBCs by Hoechst fluorescence. In agreement with the microscopy data, when the geometric mean of NBD fluorescence intensity in each population was calculated, iRBCs had significantly more fluorescence than uRBCs for all three lipid classes. This indicates a higher level of lipid internalisation in iRBCs, and therefore a higher level of flippase and/or scramblase activity (p < 0.001; **Fig 1B**). There were differences in internalisation between the lipid classes: approximately two times more NBD-PS than NBD-PC, and three times more than NBD-PE was internalised by iRBCs (**S1B Fig**). The same preference of NBD-PS > NBD-PC > NBD-PE was observed in uRBCs. Population histograms indicate that a large portion (≥50%) of the iRBC population had higher fluorescence intensity than the uRBC population for all three lipids (**S1C Fig**).

### Infected RBCs have higher scramblase and flippase activity than uninfected RBCs

Because scramblase functions independently of ATP, whereas flippase function is dependent on ATP hydrolysis, performing the assay in the presence of the ATP hydrolysis inhibitor vanadate can distinguish between flippase and scramblase activity [65]. Erythrocytes lack endocytosis, so it can be assumed that scramblase activity is responsible for the ATP-independent portion of internalisation, while flippase activity can be calculated from the difference between the total internalisation and the ATP-independent portion (*Total–Scramblase = Flippase*). A significantly higher ATP-independent NBD-lipid internalisation in iRBCs compared to uRBCs was observed in all three classes of lipid (p < 0.001; **Fig 1B**). Since scramblases not only function independently of ATP, but are also non-selective for a particular class of lipid, these data indicate that scramblase activity is significantly increased in iRBCs in comparison to uRBCs. The population data indicate that ~45–80% of iRBCs had a higher level of NBD-PS, NBD-PE, or NBD-PC than uRBCs; indicating that scramblase activity is elevated in a large portion of cells (**S1C Fig**).

Treatment with vanadate significantly decreased the mean internalisation of the most prominent lipid, NBD-PS, in both uRBCs and iRBCs, by 85% and 55%, respectively. This finding indicates that flippase activity is responsible for a significant portion of the observed NBD-PS internalisation (p < 0.001; **Fig 1B**). By subtracting the mean fluorescence intensity (MFI) of vanadate-treated cells (ATP-independent internalisation) from the MFI of untreated cells (total internalisation), we also found that iRBCs had significantly higher ATP-dependent internalisation of NBD-PS than uRBCs (**Fig 1C**), indicating an increase in flippase activity upon infection (p < 0.001). Due to the counteraction of bidirectional scramblase activity, the difference we measured between uRBCs and iRBCs likely underestimates the true difference in flippase activity. In contrast, vanadate treatment did not have a significant effect on NBD-PE (p = 0.92) or NBD-PC (p = 0.26) internalisation in iRBCs (**Fig 1B**). This finding indicates that scramblase rather than flippase is primarily responsible for internalisation of these lipids in iRBCs.

Together, these data revealed enhanced flippase activity in *P*. *falciparum*-infected erythrocytes in comparison to uninfected erythrocytes, which results in increased internalisation of NBD-PS. At the same time, incorporation of a small portion of NBD-PS and all of NBD-PE and NBD-PC in an ATP-independent manner, was also observed in iRBCs, most likely indicative of scramblase activity.

### No role for the new permeability pathway in NBD-lipid uptake

Uptake of NBD-lipids could also occur via additional mechanisms other than flippase- or scramblase-mediated movement across the bilayer. Therefore, we investigated the effect of inhibitors on lipid internalisation. To exclude that NBD-lipids were passively entering iRBCs through the New Permeability Pathway (NPP) induced by the parasite, we performed the assay in the presence of the NPP inhibitor furosemide [66]. Furosemide had no significant effect on lipid internalisation (**Fig 1D**; p > 0.05 for all comparisons), providing evidence that the NBD-lipids are not passively entering the iRBCs through these pathways, and must first flip across the membrane.

### iRBCs expend ATP to counteract increased exposure of PS in the outer membrane leaflet

**Fig 2:**
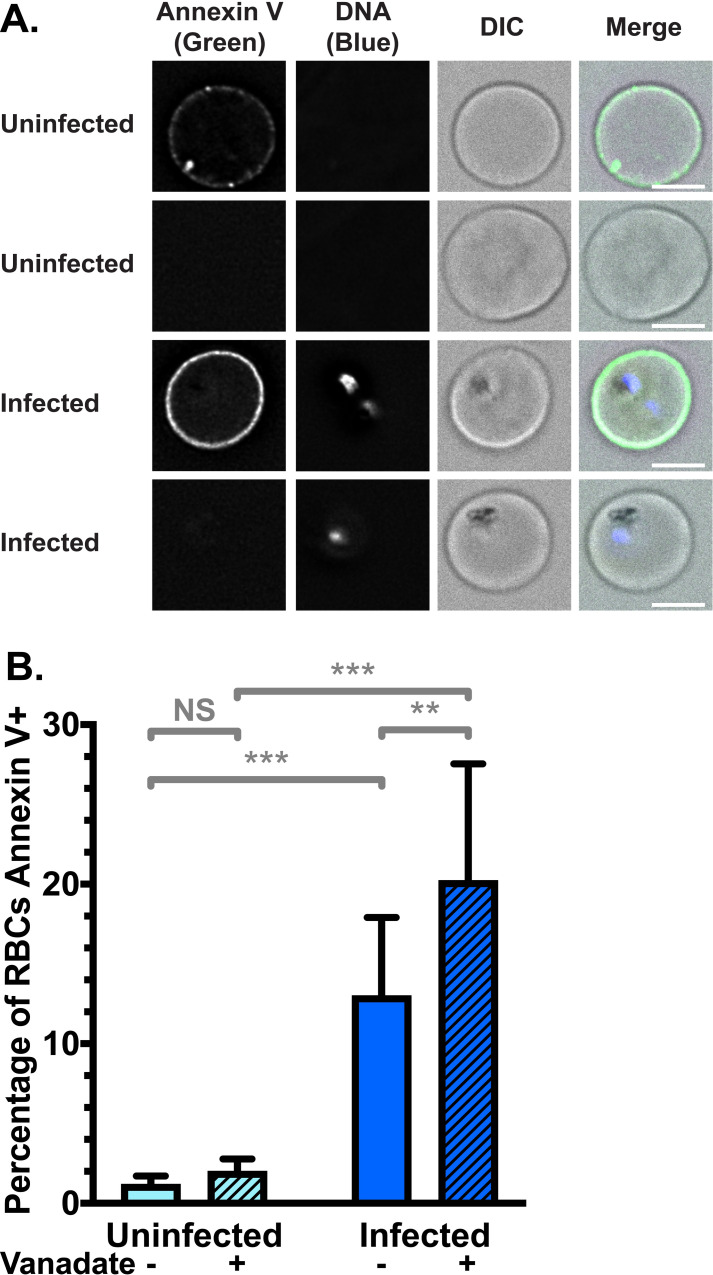
Red Blood Cell (RBC) surface expression of phosphatidylserine (PS) increases upon *Plasmodium falciparum* infection. (**A**) Example images of Annexin V positive and negative RBCs, showing heterogeneity across the population. FITC-Annexin V fluorescence was detected at 475 nm (ex)/ 525 nm (em) and Hoechst fluorescence (parasite DNA) was detected at 390 nm (ex)/ 435 nm (em). Scale bar = 4 μm. (**B**) Mean percentage (± S.D.) of RBCs exposing PS in the membrane outer leaflet, measured by FITC-Annexin V staining above background levels (no Annexin V control) in flow cytometry, with and without 0.5 mM vanadate treatment to inhibit ATP hydrolysis. NS = not significant; ** = p < 0.01; *** = p < 0.001 (ANOVA). *n* = 3 independent experiments.

To determine the proportion of PS in the outer leaflet and the outcome of a concurrent increase in both flippase and scramblase activity, we investigated PS exposure using Annexin V staining. Annexin V binds to PS when present in the membrane outer leaflet, and can be conjugated to a fluorophore such as FITC. Using deconvolution fluorescence microscopy, we found FITC-Annexin V binding was heterogeneous across the population of both uRBCs and iRBCs, with some cells highly fluorescent and others not, even amongst parasites of the same stage (**Fig 2A**). The proportion of fluorescent iRBCs was higher than the proportion of uRBCs. We therefore quantified the percentage of the population which bound FITC-Annexin V using flow cytometry analysis. We found that only 1.2% (± 0.6%) of uRBCs bound Annexin V, while 13% (± 5%) of iRBCs did (**Figs 2B** and **S3A**; p < 0.001).

Vanadate had no significant effect on the portion of uRBCs which were Annexin V-positive (2.0% ± 0.8%; p = 0.7), indicating that uRBCs do not need to expend significant amounts of energy to maintain PS asymmetry, consistent with the low level of scramblase activity we observed. In contrast, the percentage of Annexin V-positive iRBCs further increased to 20% (± 7%) when treated with vanadate (p = 0.002), indicating that iRBCs expend energy to compensate for PS exposure. These data are in agreement with the decrease of NBD-PS internalisation resulting from vanadate treatment measured in **Fig 1**; inhibiting flippase activity is therefore one plausible explanation for increased PS exposure in the outer leaflet under this condition. These results are consistent with the notion of scramblase activation in iRBCs resulting in disruption of membrane asymmetry and PS exposure in the outer leaflet. Exposure of this phagocytosis signal can at least partly be counteracted in an ATP-dependent manner. This differs to the situation in uRBCs, where very little PS exposure is observed in the outer leaflet, even with vanadate treatment.

### Calcium accumulates in intracellular parasites and occasionally in the RBC cytoplasm

Scramblase is activated by intracellular Ca^2+^, but previous reports have reached conflicting conclusions on whether this differs between uRBC and iRBC cytoplasm [58–59]. In order to investigate the underlying causes of the increase in flippase and scramblase activity between uRBCs and iRBCs, which results in PS exposure, we turned our attention to intracellular calcium. We first treated uRBCs and iRBCs with the calcium ionophore A23187, which induces calcium flux across membranes, and measured PS exposure (**S3A** and **S3B Fig**). We detected significant increases in PS exposure on both uRBCs and iRBCs (p = 0.004 and 0.02 respectively), consistent with a critical role of elevated calcium levels in increased PS exposure.

**Fig 3:**
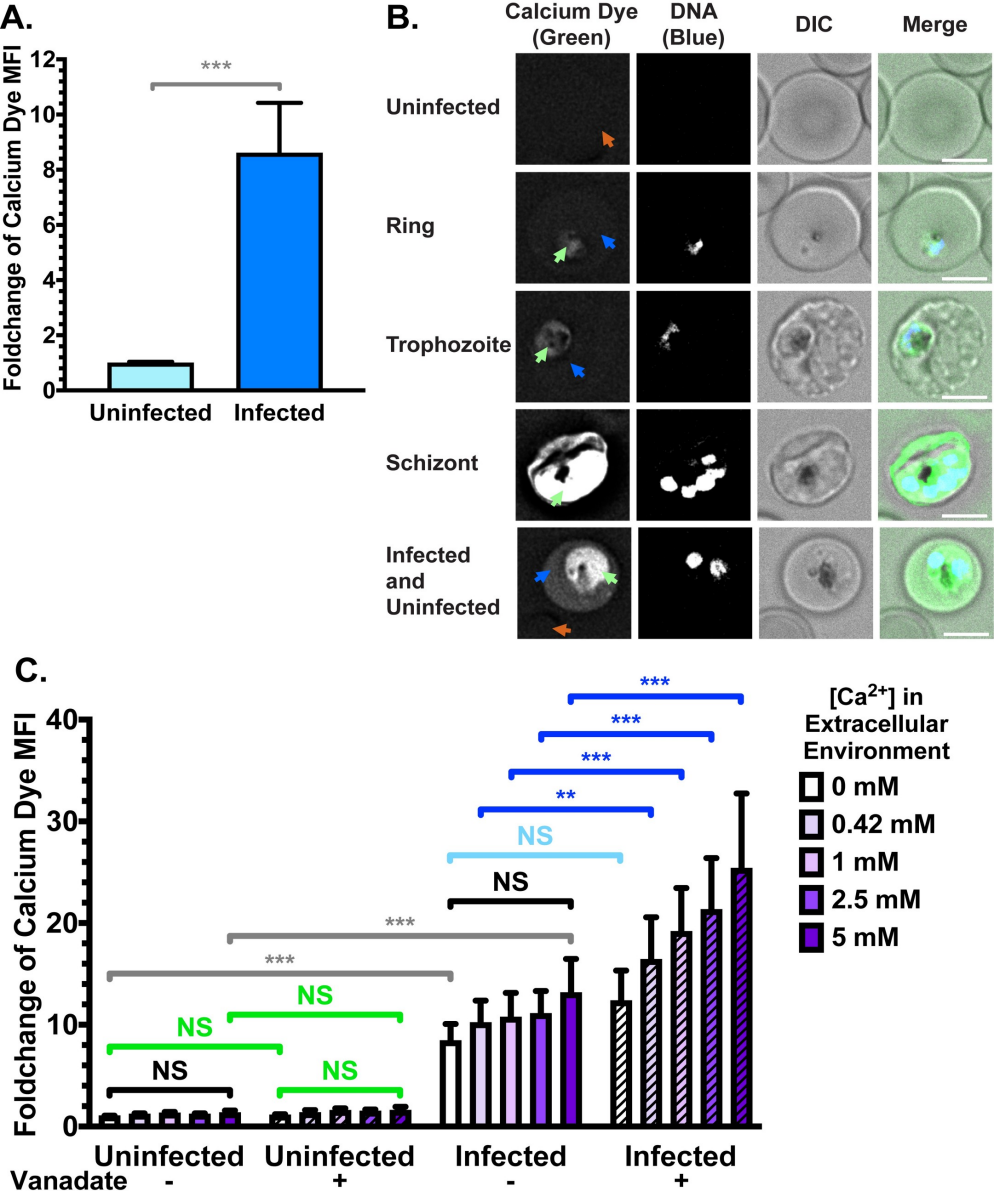
Increase of intracellular calcium upon *Plasmodium falciparum* infection. (**A**) Flow cytometry quantification of Cal520 dye mean fluorescence intensity (MFI) in uRBCs and iRBCs suspended in serum-free cell culture medium (RPMI; 0.42 mM Ca^2+^). Shown are mean values (± S.D.) *** = p < 0.001 (Mann-Whitney). *n* = 3 independent experiments. (**B**) Calcium dye fluorescence localisation by live cell deconvolution fluorescence microscopy at 37°C in clear bicarbonate-free RPMI. Calcium dye fluorescence was detected at 475 nm (ex)/ 525 nm (em) and Hoechst fluorescence (parasite DNA) was detected at 390 nm (ex)/ 435 nm (em). Shown are representative images (from top to bottom) of a uRBC, a ring-stage iRBC, a trophozoite-stage iRBC, a schizont-stage iRBC, and an iRBC representative of a portion of infected cells. Green arrows = parasite/parasitophorous vacuole; blue arrows = iRBC cytoplasm; orange arrows = uRBC cytoplasm. Scale bar = 4 μm. *n* = 3 independent experiments. (**C**) Calcium dye fluorescence intensity in uRBCs and iRBCs across different extracellular Ca^2+^ concentrations, with and without vanadate treatment to inhibit ATP hydrolysis. NS = not significant; ** = p < 0.01; *** = p < 0.001 (ANOVA). *n* = 3 independent experiments.

We next quantified the intracellular calcium content of uRBCs and late-stage (trophozoite/schizont) iRBCs using the calcium-sensitive fluorescent dye Cal520-AM [67]. The membrane-permeable dye crosses into the cell, where it is cleaved by esterases and retained inside the cell; the fluorescence intensity is proportional to the calcium concentration [68]. Cal520-AM and other calcium-sensitive AM dyes have previously been applied to uRBCs and iRBCs [67,69], supporting the notion that the RBC cytoplasm contains sufficient esterase activity to ensure this is not a rate-limiting step in fluorescence intensity. We observed significantly higher fluorescence in whole iRBCs compared to uRBCs, equalling approximately a 7.5-fold-change (± 0.9, p < 0.001) (**Fig 3A**).

In order to investigate the subcellular localisation of intracellular calcium, we imaged the fluorescence of Cal520-AM dye in live cells under physiological conditions. In agreement with the above data, we observed negligible fluorescence in uRBCs, with no specific subcellular localisation (**Fig 3B, top panel**). iRBCs were consistently more fluorescent, with the fluorescence concentrated around the part of the iRBC containing the parasite and/or parasitophorous vacuole (**Fig 3B; green arrows**). This difference was apparent regardless of parasite size and development stage, but noticeably increased in later-stage trophozoite- and schizont-stage parasites. In some cells, the RBC cytoplasm displayed a similar level of fluorescence regardless of whether they were infected (**blue arrows**) or uninfected (**orange arrows**). However, some iRBCs displayed an increased fluorescence in the iRBC cytoplasm compared to uRBCs from the same culture (**bottom panel**). This proportion (~60%) was roughly consistent with the portion of iRBCs which displayed higher scramblase activity (**S1C Fig**). However, the observed changes were small, and alone may not account for the observed increases in scramblase function. RBCs infected with trophozoite and schizont stage parasites were more likely to display higher cytoplasmic calcium than ring-stage iRBCs, but many late-stage iRBCs still maintained comparable host cytoplasm fluorescence to uRBCs (**Fig 3B, middle panels**).

Together, these data indicate that many iRBCs maintain low Ca^2+^ levels in the host cytoplasm, but some iRBC have elevated levels in the host cell cytoplasm. This portion of iRBCs may represent older RBCs which have a reduced capacity to regulate Ca^2+^ homeostasis, or could result from transient increases while Ca^2+^ crosses the cytoplasm to the parasitophorous vacuole and parasite.

### Maintenance of calcium equilibrium in iRBCs requires ATP hydrolysis

Since these experiments were conducted in serum-free cell culture media (RPMI, containing 0.42 mM Ca^2+^), we tested whether varying the extracellular Ca^2+^ concentration influences the intracellular concentration by suspending RBCs in Ringer Solution containing various Ca^2+^ concentrations (from 0 mM to 5 mM). The difference between uRBCs and iRBCs was evident at all tested concentrations of calcium in the extracellular environment (**Fig 3C; grey bars**; p < 0.001 for all comparisons). Furthermore, in both uRBCs and iRBCs, no significant changes to calcium dye fluorescence were evident when varying the extracellular calcium concentration (**Fig 3C; black bars**; p > 0.05 for all comparisons), indicating that the cells can maintain a fairly constant intracellular calcium level despite changes to the extracellular environment, at least within our 60-minute incubation in glucose-containing media.

We then tested the ATP-dependency of maintaining the Ca^2+^ gradient by adding the ATP hydrolysis inhibitor, vanadate. In uRBCs, the addition of vanadate did not significantly change the intracellular fluorescence (**Fig 3C; green bars**; p > 0.05 for all comparisons), indicating that uRBCs do not need to expend large amounts of energy to maintain low intracellular calcium levels. In contrast, iRBCs treated with vanadate were unable to maintain the intracellular calcium levels in the same way as untreated cells. A correlation between extracellular calcium concentration and increases in cellular calcium dye fluorescence was observed in vanadate-treated iRBCs (**Fig 3C; dark blue bars;** p = 0.006 for 0.42 mM; p < 0.001 for 1 mM, 2.5 mM, 5 mM). No significant increase was observed in vanadate-treated iRBC samples with no calcium in the extracellular environment (**Fig 3C; light blue bar**; p = 0.22), providing evidence that energy is required to counteract Ca^2+^ influxes from the extracellular environment.

Since treatment with the calcium ionophore A23187 significantly increased PS exposure (**S3B Fig**), we wanted to ensure efficient calcium influx upon ionophore treatment. No effect was detected in uRBCs or iRBCs when calcium was absent in the extracellular medium (**S3C Fig,** p = 0.87 and p = 0.85 respectively). As expected, A23187 treatment resulted in increase in calcium dye fluorescence in all samples with Ca^2+^ in the extracellular media (p < 0.05 for uRBCs and p < 0.001 for iRBCs across all concentrations).

Together, these data confirm that iRBCs contain more calcium than uninfected erythrocytes, and that energy has to be expended to prevent further increases in intracellular calcium levels. Maintenance of intracellular calcium is therefore a second mechanism by which the iRBC expends energy to decrease PS exposure, in addition to flippase activity. In comparison, uRBCs maintain low intracellular calcium levels, and low PS exposure, without expending large amounts of energy.

### iRBCs have a lower cholesterol content in the RBC membrane

**Fig 4:**
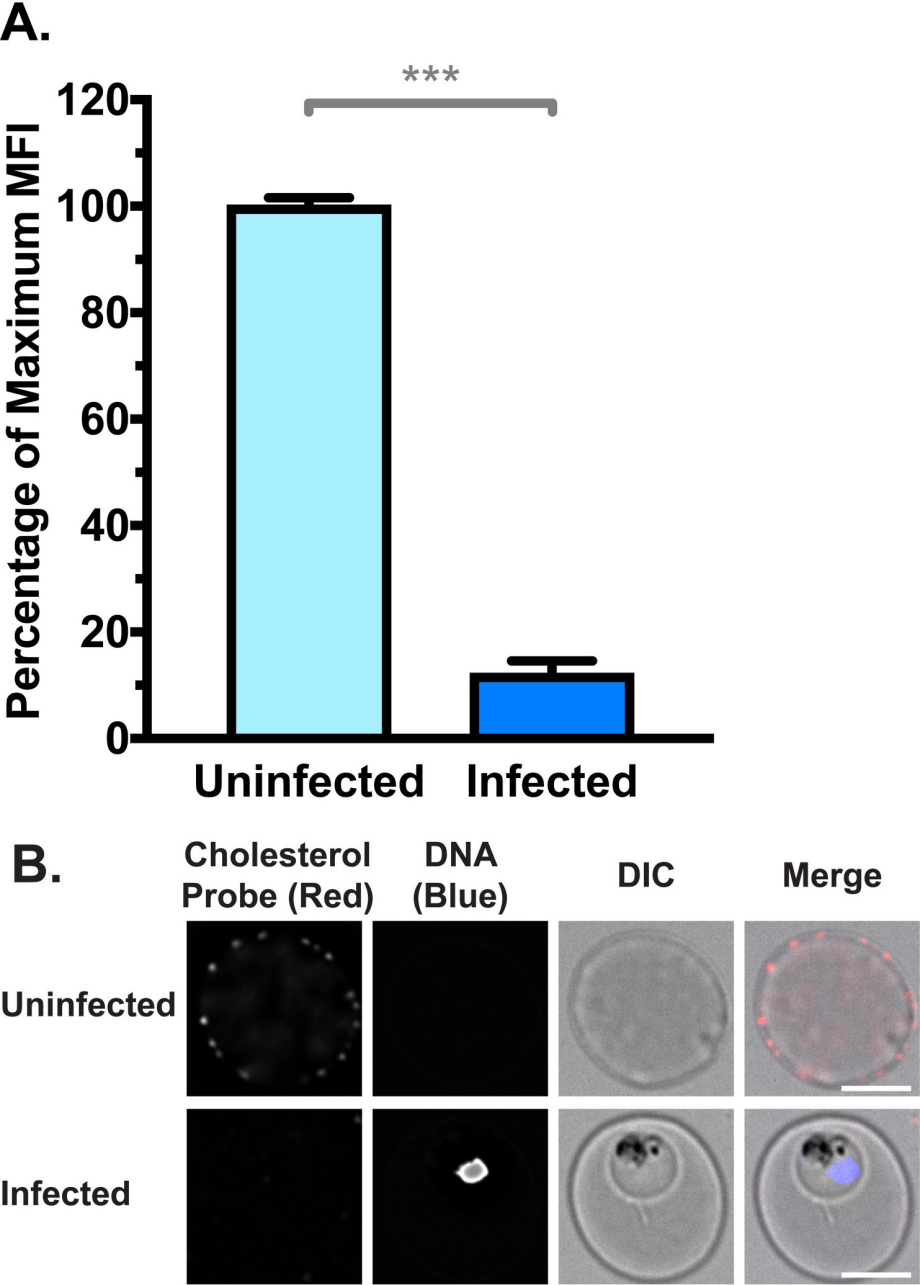
RBC Membrane cholesterol is depleted in iRBCs, measured by binding of a fluorescent cholesterol probe θ-D4-mCherry. (**A**) Normalised mean fluorescence intensity (MFI) of cholesterol probe measured by flow cytometry. Shown are mean values (± S.D.), *** = p < 0.001 (Mann-Whitney). *n* = 3 independent experiments (**B**) Cholesterol probe from (A) visualised by deconvolution fluorescence microscopy. mCherry fluorescence was detected at 575 nm (ex)/ 626 nm (em) and Hoechst fluorescence (parasite DNA) was detected at 390 nm (ex)/ 435 nm (em). Scale bar = 4 μm.

Membrane cholesterol is reported to repress the function of scramblase enzymes [70,71]. Previous reports have also proposed that iRBC membranes may be depleted of cholesterol, due to sequestration by the intracellular parasite, coupled with a lack of *de novo* synthesis [72–75]. Therefore, we examined the cholesterol content of uRBC and iRBC membranes with a cholesterol-binding probe (θ-D4) conjugated to the fluorophore mCherry [76]. Unpermeabilised iRBCs had approximately a 10-fold decrease in probe fluorescence compared to uRBCs, indicating a significant depletion of cholesterol from the iRBC membrane (**Fig 4A**; p < 0.001). The difference between uRBCs and iRBCs was also evident at the single cell level, by examination under a fluorescence microscope (**Fig 4B**).

### iRBCs are recognised and phagocytosed by monocytes

**Fig 5:**
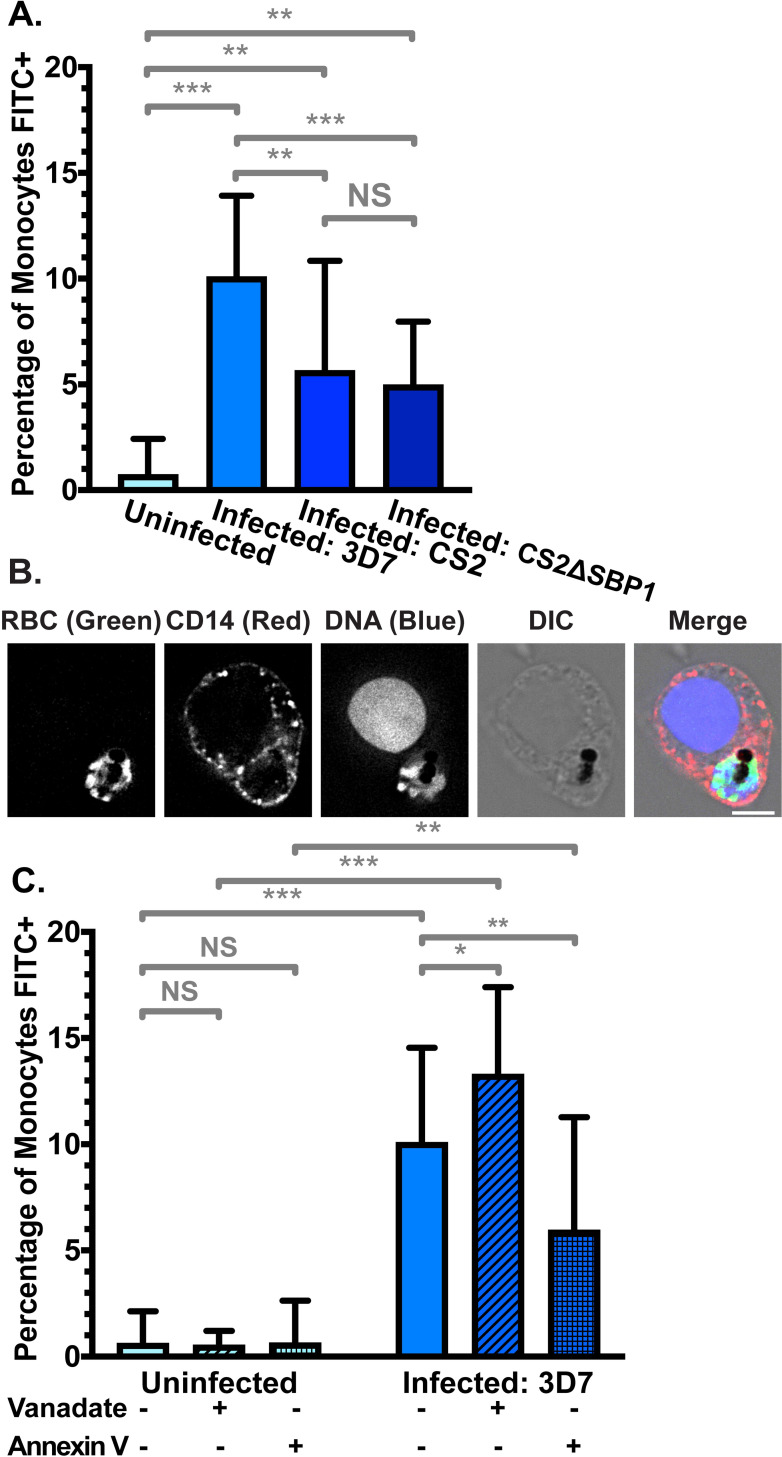
Phagocytosis of FITC-stained RBCs by primary CD14+ monocytes. (**A**) Phagocytosis of uRBCs and iRBCs parasitised by *P*. *falciparum* 3D7 wildtype, CS2 wildtype, and skeletal-binding protein knock-out (CS2ΔSBP1) parasite strains. Shown is the mean percentage (± S.D.) of CD14+ (PerCP) monocytes with at least one phagocytosed FITC-stained RBC. NS = not significant; ** = p < 0.01; *** = p < 0.001 (ANOVA). *n* ≥ 3 independent experiments for uRBC, 3D7 iRBC, and CS2ΔSBP1 iRBC; *n* = 2 independent experiments for CS2 wildtype iRBC (**B**) Representative monocyte from (A) with a phagocytosed 3D7 iRBC. FITC fluorescence was detected at 475 nm (ex)/ 525 nm (em), PerCP fluorescence (anti-CD14) was detected at 475 nm (ex)/ 679 nm (em), and Hoechst fluorescence (DNA) was detected at 390 nm (ex)/ 435 nm (em). Scale bar = 6 μm. (**C**) Phagocytosis of uRBCs and 3D7 iRBCs, with and without 0.5 mM vanadate treatment to inhibit ATP hydrolysis, or 5 μg/mL Annexin V to block exposed PS. NS = not significant; * = p < 0.05; ** = p < 0.01; *** = p < 0.001 (ANOVA). *n* ⩾ 3 independent experiments.

To investigate whether PS exposure enhances phagocytosis, we compared uptake of RBCs by CD14+ primary monocytes isolated from buffy coat. We measured phagocytosis under stringent conditions by i) limiting incubation to only 30 minutes, ii) applying a relatively low ratio of iRBCs to monocytes (20:1) and iii) not treating RBCs with any opsonising factors, such as antibodies or complement. In line with the low level of PS exposure, phagocytosis of uRBCs was very low, with fewer than 1% of monocytes phagocytosing a uRBC (**Fig 5A**). In contrast, phagocytosis of purified iRBCs infected with the 3D7 parasite strain was significantly higher (p < 0.001), with 10% (± 4%) of monocytes phagocytosing at least one iRBC).

Phagocytosis of iRBCs can also occur via the parasite-encoded RBC surface protein *Pf*EMP1 binding to CD36 on monocytes [77,78]. To examine the influence of CD36-mediated *Pf*EMP1 binding on phagocytosis, we examined RBCs infected with the *P*. *falciparum* strain CS2, which expresses VAR2CSA, a *Pf*EMP1 variant which binds to chondroitin sulfate A (CSA) instead of CD36 [79]. Phagocytosis of CS2 wildtype iRBCs was significantly higher than uRBCs (6% ± 5%; p = 0.004), confirming that substantial phagocytosis can occur in the absence of CD36 binding by *Pf*EMP1 (**Fig 5A**), though phagocytosis was reduced compared to the 3D7 strain iRBCs (p = 0.0095). This difference between 3D7 and CS2 might be attributable to phagocytosis mediated by *Pf*EMP1 binding to CD36 and not PS exposure.

To provide independent evidence and further exclude other *Pf*EMP1 variants playing a role in phagocytosis, we also examined a CS2 cell line where skeletal-binding protein 1 (SBP1) is deleted (CS2ΔSBP1) [80]. In this line, the central trafficking hub, Maurer’s clefts, are dysfunctional and transport of *Pf*EMP1 to the RBC membrane is disrupted. Similar to RBC infected with the CS2 parental parasite line, CS2ΔSBP1 iRBC are also phagocytosed by monocytes (5% ± 3%; p = 0.004 compared to uRBCs). Phagocytosis remained similar to the CS2 parental strain, indicating that potential additional defects in protein trafficking to the RBC surface do not further affect phagocytosis (p = 0.72). In all experiments with iRBCs, monocytes with phagocytosed iRBCs were clearly visible using fluorescence microscopy, where the FITC-stained RBC, parasite DNA, and parasite haemozoin crystal were apparent within the monocyte (**Figs 5B** and **S4**). These experiments clearly demonstrate that phagocytosis of iRBCs still occurs in the absence of *Pf*EMP1-mediated CD36 binding, in good agreement with a central role of PS exposure in phagocytosis.

In order to investigate the impact of increased PS exposure on phagocyte recognition, we additionally pre-treated RBCs with vanadate to inhibit the action of flippase and calcium efflux pumps. Phagocytosis of uRBCs was not significantly altered, in line with the very low increase in PS exposure we observed with vanadate treatment (**Fig 5C**, p = 0.71). However, phagocytosis was 32% higher when *P*. *falciparum* 3D7 iRBCs were pre-treated with vanadate (p = 0.01), indicating that the iRBCs expend energy on processes that ultimately reduce phagocytosis. Given the short duration of the experiment and the potential impact of vanadate on protein synthesis and transport, other monocyte recognition signals on the iRBC surface (such as *Pf*EMP1) are unlikely to increase at the same time.

Finally, we examined the effect of directly blocking exposed PS using Annexin V. Phagocytosis of uRBCs was not significantly different in the presence of Annexin V (**Fig 5C**; p = 0.75), but when exposed PS was shielded by Annexin V, we detected a significantly lower rate of phagocytosis in 3D7 iRBCs, representing a decrease of 41% compared to the absence of Annexin V (p = 0.043). This provides independent evidence that exposed PS is an important signal for phagocytosis of iRBCs by monocytes. Together, we demonstrated that PS exposure on the outer leaflet of the red cell membrane leads to phagocytosis of *Plasmodium falciparum* infected RBCs.

### NBD-Phospholipid internalisation by isolated parasites

**Fig 6:**
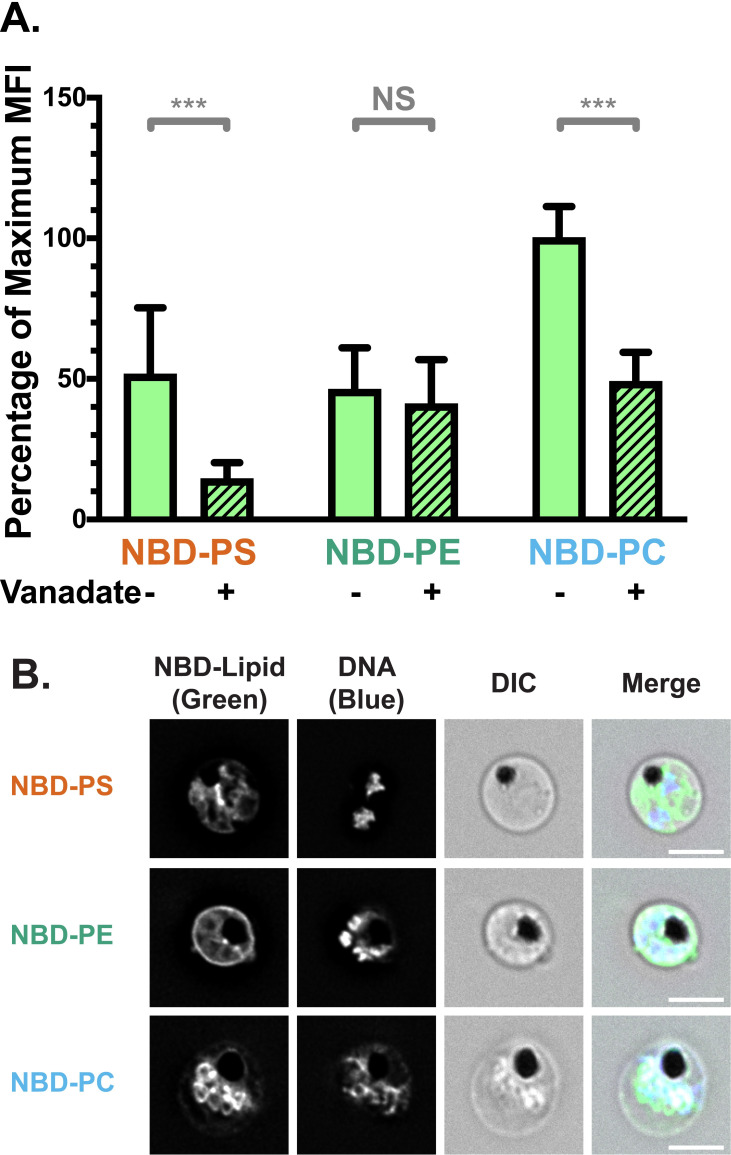
Saponin-isolated parasites internalise NBD-lipids at the parasite plasma membrane. (**A**) NBD-lipid internalisation, measured by increase in NBD mean fluorescence intensity (MFI) in flow cytometry after extraction of lipids remaining in the outer layer. Cells were treated with vanadate in calcium-free media to measure only the ATP-independent portion of internalisation. Shown are mean values (± S.D.), NS = not significant; *** = p < 0.001 (ANOVA). *n* = 3 independent experiments. (**B**) Subcellular localisation of NBD-PS (top), NBD-PE (centre), and NBD-PC (bottom) in saponin-isolated parasites from (A) after internalisation, visualised by deconvolution fluorescence microscopy. NBD-lipid fluorescence was detected at 475 nm (ex)/ 525 nm (em) and Hoechst fluorescence (parasite DNA) was detected at 390 nm (ex)/ 435 nm (em). Scale bar = 4 μm.

We also wanted to examine if lipid internalisation occurs at the parasite plasma membrane. To test this, the host cell and parasitophorous vacuole membranes were lysed with saponin. We then repeated the lipid internalisation assay (**Fig 1**) on the saponin-isolated parasites. Internalisation of NBD-PS, NBD-PE, and NBD-PC was observed in the parasites (**Fig 6A**). NBD-PC rather than NBD-PS showed the highest level of internalisation at the parasite plasma membrane, which contrasted to the pattern observed at the RBC plasma membrane (**S2B Fig**).

Similar to the host RBCs, NBD-PS internalisation was significantly reduced by treatment with vanadate, revealing that 72% of internalisation required the hydrolysis of ATP (p < 0.001). This is consistent with the existence of an ATP-dependent flippase at the parasite plasma membrane. There was no significant decrease in NBD-PE internalisation (14%; p = 0.07). NBD-PC internalisation decreased by 52% when treated with vanadate (p < 0.001). Given that most flippase enzymes do not utilise PC as a substrate, this is likely due to other, yet uncharacterised ATP-dependent lipid uptake pathways at the parasite membrane.

Since saponin lysis can leave fragments of the host cell membrane attached to the parasite, we also analysed the treated cells under the fluorescence microscope (**Figs 6B** and **S5**). While some parasites still had host cell membrane or PVM fragments surrounding them, all observed fluorescence was associated with the parasite; thus, all NBD-lipids were successfully extracted from any remaining host cell membrane remnants, and the assay measured only the uptake at the parasite plasma membrane. We also observed a diffuse NBD-fluorescence within the parasite, potentially indicating further uptake and metabolism of the lipid analogues by the parasite.

## Discussion

**Fig 7:**
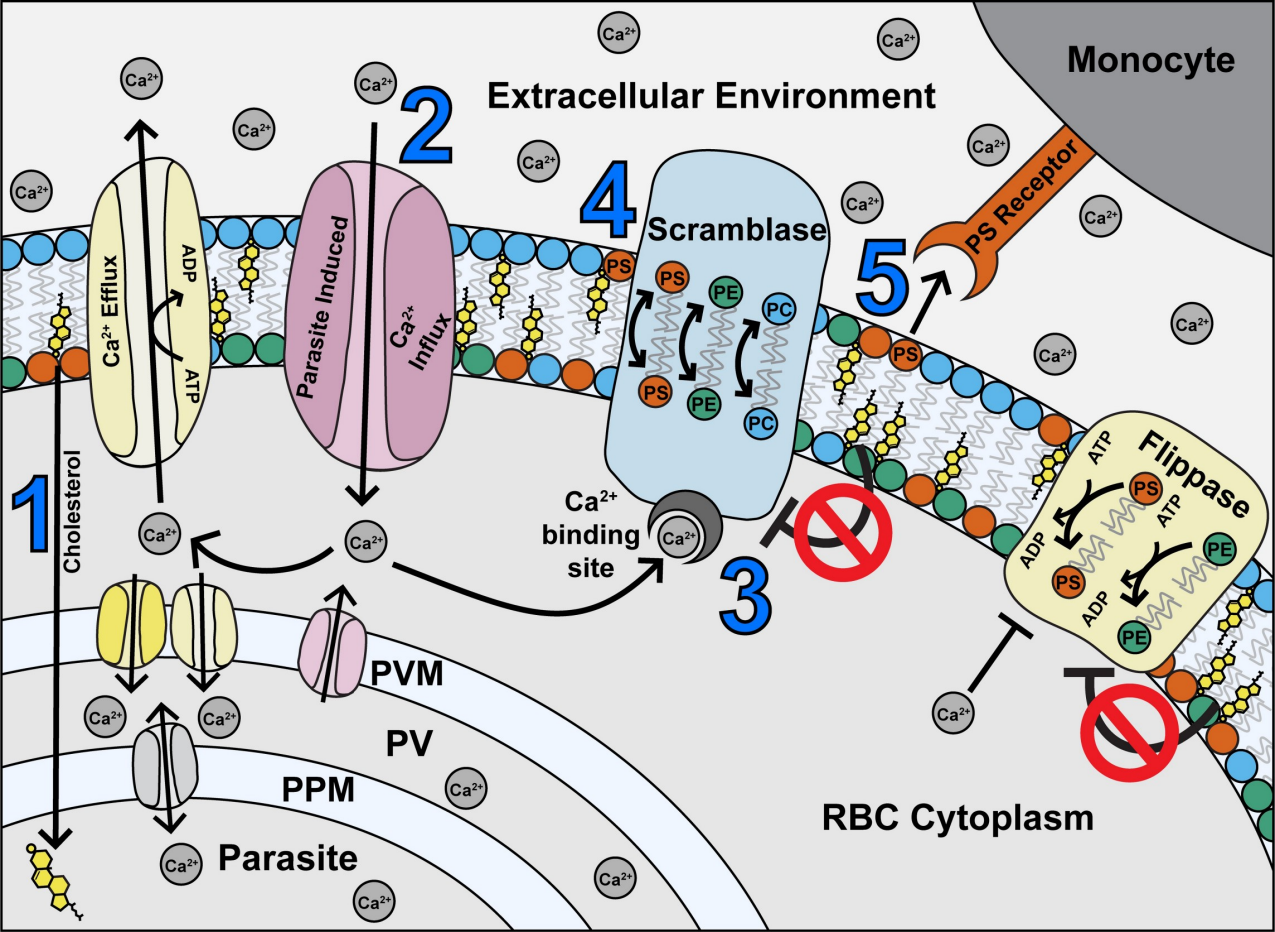
Proposed model of maintenance of phospholipid asymmetry in a *Plasmodium falciparum*-infected erythrocyte, based on the findings of this study. (**1**) The parasite sequesters cholesterol from the host RBC, leaving the RBC membrane depleted of cholesterol (**2**) Parasite infection also causes an increase in Ca^2+^ influx to the RBC from the external environment, and potentially by leakage from the PV (**3**) Ca^2+^ ions activate the scramblase enzymes, while repression by cholesterol is removed (**4**) Scramblase activation results in exposure of PS in the outer membrane leaflet (**5**) Exposed PS acts as a recognition signal for monocytes. Most iRBCs are rescued from phagocytosis by compensatory mechanisms (in yellow) including efflux and/or parasite uptake of Ca^2+^ ions by host or parasite channels, and flippase-mediated internalisation of PS. PS = phosphatidylserine; PE = phosphatidylethanolamine; PC = phosphatidylcholine; PPM = parasite plasma membrane; PV = parasitophorous vacuole; PVM = parasitophorous vacuole membrane; ATP = adenosine triphosphate; ADP = adenosine diphosphate.

In this study, we present a series of interconnected relationships between *Plasmodium falciparum* parasites and their host RBCs. While some of the induced changes to the host cell promote parasite survival, some can lead to its downfall. Based on the above data, we present a model (**Fig 7**) in which parasite-induced changes to the RBC membrane, such as (**1**) depleted cholesterol and (**2**) increased permeability resulting in Ca^2+^ influx, (**3**) activates scramblase enzymes. In turn, this results in (**4**) altered membrane asymmetry and PS exposure, and (**5**) recognition and phagocytosis by monocytes, as well as increased flippase activity.

We have also shown that the iRBCs expend energy to compensate for the higher level of PS exposure, preventing some iRBCs from being phagocytosed. This is evidenced by the increase in both PS exposure and PS-dependent monocyte phagocytosis when iRBCs are treated with vanadate to inhibit ATP hydrolysis. Previous work has established that iRBCs have a significantly higher demand for glucose and ATP than their uninfected host cells [81,82]. While this energy is used for a wide range of cellular processes, we found that two ATP-dependent processes are used to compensate for PS exposure (**Fig 7, in yellow**). The first process is to maintain the (albeit elevated compared to uRBCs) intracellular Ca^2+^ equilibrium constant through active efflux into the extracellular environment and/or by uptake into the parasitophorous vacuole (PV) and parasite. A candidate protein for this activity is the endogenous RBC Plasma Membrane Ca^2+^ ATPase (PMCA), which is active in iRBCs [83], possibly along with parasite-encoded proteins. The second mechanism we propose is an increase in flippase activity to reinternalize PS which has become exposed. Previous studies in other cell types have shown that apoptotic cells and activated platelets must repress flippase activity concurrently with activating scramblase, in order to expose PS [84,85]. Since our data indicates that flippase is still active in iRBCs, we expect that this enzyme is compensating for some PS exposure. It has previously been reported that a certain threshold of PS exposure is required for phagocytic recognition of apoptotic cells by macrophages [86], and thus these regulatory mechanisms may keep PS exposure below this threshold in most iRBCs. However, despite these mechanisms, a portion of iRBCs (~13%) fail to avoid PS exposure, and some thus succumb to phagocytosis. In contrast, uRBCs need to spend only a negligible amount of energy to avoid PS exposure or phagocytosis. This is likely because scramblase activity is repressed, and apparently remains so even in the presence of high extracellular Ca^2+^ concentrations.

A large portion of iRBCs in our study (~40%) displayed similar calcium dye fluorescence in the RBC cytoplasm as compared to uRBCs, indicating that many iRBCs do not have elevated Ca^2+^ in the host cytoplasm, as supported by earlier studies [57,59]. These studies were in contrast to Zipprer *et al*. [58], who reported that Ca^2+^ was higher in the RBC cytoplasm across the population; however, their assay buffer did not contain any glucose or ATP, which leads to swift Ca^2+^ influx in iRBCs through a channel which is not otherwise active in glucose-containing media [87].

We also observed a population of iRBCs (~60%) which had elevated Ca^2+^ concentrations in the iRBC cytoplasm; while there was some correlation with increasing parasite development, this trend was not consistent. This high Ca^2+^ population may represent older RBCs which have a lower capacity to extrude Ca^2+^ [88]. Previous studies have also found that when RBCs are stored, flippase activity decreases over time, and both scramblase activity and PS exposure increase [89]. This and other studies have also concluded that younger RBCs had higher flippase activity than older (more dense) RBCs in blood that has not been stored [76,89]. Older RBCs may therefore be unable to cope with the increased strain imposed by the parasite, even though their capacity to maintain Ca^2+^ homeostasis is not compromised prior to infection. Separation of senescent and non-senescent erythrocytes in combination with *P*. *falciparum* infections may permit a systematic analysis of combined and distinct effects of RBC ageing on PS exposure, flippase, scramblase, and Ca^2+^ regulation [14, 88–90]. The elevated Ca^2+^ content is likely the cause for scramblase activation in older RBCs, further emphasising the importance of Ca^2+^ regulatory mechanisms for parasite survival. However, it has been reported that intracellular calcium in normal uRBCs must increase more than 100 times in order to activate scramblase enzymes [52]; the small changes we observed may not account for the high degree of scramblase activity. Furthermore, we detected a concurrent increase in flippase activity in iRBCs, although Ca^2+^ has been reported to inhibit flippase at concentrations lower than that required to activate scramblase [52,76]. Therefore, we do not think our results can be explained by an increase in RBC cytoplasmic Ca^2+^ alone. Based on our data showing depletion of cholesterol from the iRBC membrane, we propose that this effect additionally contributes to scramblase activation. Arashiki *et al*. [70] have previously demonstrated that membrane cholesterol is a potent inhibitor of scramblase in RBCs, and its depletion can result in exposure of PS even at very low calcium concentrations. Increased scramblase activity has also been observed in RBCs with both naturally occurring and artificially induced cholesterol depletion [71]. We therefore propose that the parasite-induced depletion of membrane cholesterol removes the inhibitory effect on scramblase (**Fig 7**), and allows activation without large increases in cytoplasmic Ca^2+^.

Incidentally, the increased flippase activity observed in iRBCs may also be explained by the parasite-induced cholesterol depletion. Morrot *et al*. [91] have previously demonstrated that flippase activity increases with cholesterol depletion. They proposed that this was due to the higher fluidity of cholesterol-depleted membranes, which could affect the turnover rate of the flippase enzymes. In their study, altering the cholesterol:phospholipid ratio in RBCs did not change the amount of PS or PE which flipped into the inner leaflet at end point–only the time taken to reach the plateau. Therefore, the cholesterol depletion likely influences the activity of the enzymes resulting in PS exposure, rather than being the direct cause. It is noteworthy that while the increased flippase activity would compensate for a portion of scramblase activity, some iRBCs clearly fail to re-internalise all exposed PS, and thus are recognised by phagocytes. At present, it is difficult to determine if the increase in flippase activity occurs across the population or only in a portion of iRBCs. Since our assay was performed in a Ca^2+^ free media, it is unlikely that vanadate treatment itself activates scramblase within this experiment.

We have also demonstrated that lipid internalisation occurs at the parasite plasma membrane. Energy-dependent internalisation of NBD-PS is consistent with a flippase protein present in this membrane. Several P4-type ATPases have been identified in the *P*. *falciparum* genome, of which several have been categorised as flippases based on predicted homology. Of these, ATP2 (PF3D7_1219600) and ATP8 (PF3D7_1223400) are predicted to be essential, or for parasite strains with mutations in these genes to have severe growth defects, according to a genome-wide *piggyBac* insertion mutagenesis screen [92]. Experimental genetics in the murine malaria model *P*. *berghei* established that the orthologous genes PBANKA_143480 and PBANKA_143830 are refractory to targeted gene deletion, and the corresponding mCherry-fusion proteins localise at or near the parasite plasma membrane [93]. Other potential lipid-flipping proteins localised within the parasite interior [93]. The energy-dependent internalisation of NBD-PC could be mediated by a different uptake pathway, as most flippases do not utilise PC as a substrate [20]. However, no studies to date have looked at the substrate specificity of the parasite’s flippase, so this remains an open question. In the light of our findings, it would be interesting for future studies to determine the substrate specificity and cholesterol/Ca^2+^ sensitivity of these proteins. A candidate *Plasmodium* scramblase protein (PF3D7_1022700) has recently been identified and characterised, and could be responsible for energy-independent lipid internalisation to the parasite observed in this study [94 [pre-print]].

Microscopic analyses revealed that NBD-lipids moved beyond the parasite plasma membrane and into the parasite regardless of whether the lipids were added to intact RBCs or saponin-isolated parasites, potentially indicating pathways of host phospholipid uptake by the parasite. Although the parasite is capable of synthesising most, if not all, phospholipids *de novo* [95,96], the ability to scavenge them from the host cell membrane may be advantageous to parasite development and replication. For instance, it may be easier for the parasite to scavenge minor phospholipids, including those with specific tail groups. Wein *et al*. [95] have demonstrated marked differences in synthesis between specific subclasses, which could be related to both parasite need and synthesis capacity. The parasite may also modify the NBD-lipids after uptake according to its own lipid requirements and metabolic state, and thus the fluorescence observed within the parasite may partly represent an altered product rather than the original lipid. The measured net effect of high lipid internalisation from the RBC plasma membrane would remain similar, irrespective of the metabolic fate in the growing parasite.

The mechanism we have elucidated in this study might have broader physiological relevance and consequences for the disease outcome. The parasite seems to have optimised what is possible within the constraints of host-pathogen co-evolution: the parasite requires Ca^2+^ influx and cholesterol scavenged from the host plasma membrane to survive, but these requirements perturb the host cell and trigger responses like scramblase activation. Within a certain range this can be compensated by increased activity of the ATP dependent host flippase to prevent excessive PS exposure. However, if the compensatory mechanisms are compromised (i.e. RBC senescence or chemical perturbation), the balancing act collapses leading to PS exposure and phagocytosis of the parasitised RBC through monocytes. It is tempting to speculate about the broader implications for the parasites: this mechanism could explain the capability of *P*. *falciparum* to survive in mature RBCs, whereas *P*. *vivax* and *P*. *ovale* have a strong preference for invading reticulocytes [97]. At the same time, the phagocytosis of a subpopulation of iRBCs might not only benefit the host by slowing down the progress of infection, but also ensure the survival of the host, hence increasing the time that is available for disease transmission. Further research is needed to substantiate these potential implications.

Interestingly, a genome-wide association study uncovered that mutations in the PMCA pump, which is an excellent candidate for calcium efflux in our model, are associated with protection from severe malaria [98]. We hypothesise this protective effect seen in the epidemiological study may at least be partially explained by the calcium extrusion activity of this pump to maintain low RBC cytosolic Ca^2+^, therefore minimising scramblase activation, PS exposure, and ultimately phagocytic clearance. This association with protection from severe disease is a prime example for the delicate balance of this parasite/host interaction: small disruptions to the regulatory systems at any point in the network may tip the scales against the parasite.

It may also be possible to exploit parts of this pathway as a drug target. We have demonstrated that iRBCs are far more susceptible to interference with these regulatory mechanisms than uRBCs. We have used vanadate as a proof-of-concept compound to show that treated iRBCs are more vulnerable to PS exposure and phagocytic recognition than treated uRBCs. Evidently, a potential drug candidate would need to be far more specific. There is potential to design a tailored compound that would leave ring-stage iRBCs vulnerable to macrophage recognition in the spleen, as these early stages of parasites still circulate throughout the bloodstream. Invading merozoites cause a temporary spike in RBC cytoplasmic Ca^2+^, which is quickly corrected (within minutes), presumably by PMCA [99]. If the cell was unable to correct themselves by expelling excess Ca^2+^, PS would be exposed on circulating iRBCs, which could subsequently be phagocytosed by splenic macrophages, while leaving uRBCs unaffected, because they are protected by their generally low Ca^2+^ permeability.

Together, this report elucidates the mechanisms and events necessary for the maintenance and collapse of membrane asymmetry in RBCs infected with *Plasmodium falciparum*, and highlights the importance of this pathway to parasite survival.

## Methods

### Ethics statement

All relevant aspects of this study were approved by the Australian National University’s Human Ethics Committee, procedures HEC2017/351 and HEC2016/317. Human red blood cell and serum were kindly provided by the Australian Red Cross Blood Service (“Lifeblood”). Donor consent was obtained as part of the donation process.

### Parasite culture

*P*. *falciparum* parasites were maintained under routine culture conditions in red blood cells and RPMI 1640-Hepes with Glutamax, supplemented with 10 mM D-glucose, 480 μM hypoxanthine, 20 μg/mL gentamicin, 0.375% (w/v) ALBUMAX II, and 2.5% v/v heat-inactivated human serum [100]. All experiments used 3D7 wildtype parasites except where otherwise specified. Cultures were double synchronised with 5% w/v D-sorbitol in the days prior to experiments, to obtain majority late stage (trophozoite/schizont) iRBCs [101]. All experiments were performed at least three times on separate days except where otherwise indicated, in addition to containing technical replicates within the experiment. RBCs from different donors were pooled to account for variations between individuals, and were used within three weeks of donation. Each phagocytosis experiment used monocytes isolated from a different batch of blood, with a variety of donors used for different experiments.

### Fluorescence microscopy

Images were collected and deconvoluted on a Deltavision Deconvolution microscope at 1000x magnification, with a resolution of 0.067 μm per pixel. All images were collected under normal atmospheric conditions (in appropriate buffers) and at ambient temperature, except where otherwise specified. NBD-lipid, FITC-Annexin V, Cal520, and FITC fluorescence were detected at 475/28 nm excitation and 525/48 nm emission. Hoechst fluorescence (nucleic acid) was detected at 390/18 nm and ex/ 435/48 nm em. mCherry fluorescence was detected at 575/25 nm ex and 626/45 nm em. PerCP-anti-CD14 fluorescence was detected at 475/28 nm ex and 679/34 nm em.

Within each experiment, images were collected under the same exposure conditions (without binning) and converted to TIFF files under the same brightness and contrast settings. Individual cells were cropped from larger images with Fiji ImageJ. No other manipulations were performed.

### Flow cytometry

For quantification, events were measured on a LSR II Flow Cytometer unless otherwise specified. NBD-lipid (Avanti Polar Lipids), FITC-Annexin V (Biolegend), Cal520 (Abcam), and FITC (Sigma) fluorescence were detected at 488 nm ex/ 530 nm em. Hoechst 33342 (Thermo Fisher) fluorescence was detected at 410 nm ex/ 450 nm em. PerCP-anti-CD14 (Miltenyi Biotec) was detected at 488 ex/ 670 nm em. Data were initially processed using FlowJo. RBCs, saponin-isolated parasites, or monocytes were gated on FSC and SCC. iRBCs (positive) and uRBCs (negative) were differentiated by Hoechst fluorescence. Saponin-isolated parasites were gated on positive Hoechst fluorescence, and monocytes were gated on positive Hoechst and PerCP-anti-CD14 fluorescence. The geometric mean of fluorescence intensity (mean fluorescence intensity, MFI) and/or the percentage of each population (e.g. FITC positive) was calculated with FlowJo. Background fluorescence was subtracted based on unstained controls (buffer or solvent only). Except where otherwise specified, MFI data was normalised between experiments by setting the fluorescence of untreated uRBCs to 1 or 100%.

### Statistics

Except where otherwise specified, data were analysed in Prism 8 using one-way or two-way ANOVA with corrections for the false discovery rate using the two-stage setup method of Benjamini *et al*. [102]. Numerical data presented on graphs are available in **S1 Data**.

### Lipid internalisation assay

Lipid internalisation assays were performed according to the methods of Yabas *et al*. [64] with some modifications similar to Arashiki *et al*. [23]. A culture containing uRBCs and 5–10% late-stage (trophozoite/schizont) iRBCs was washed twice in PBS with 10 mM D-Glucose (PBS-G). RBCs were pre-treated with 100 μM Furosemide (Sigma) or solvent control (0.06% v/v methanol) for 5 minutes, and then 0.5 mM sodium orthovanadate (Sigma) or the corresponding volume of buffer only, and incubated for 5 minutes.

All lipids were sourced from Avanti Polar Lipids. 5 μM of NBD-PS (1-palmitoyl-2-{6-[(7-nitro-2-1,3-benzoxadiazol-4-yl)amino]hexanoyl}-sn-glycero-3-phosphoserine), NBD-PE (1-palmitoyl-2-{6-[(7-nitro-2-1,3-benzoxadiazol-4-yl)amino]hexanoyl}-sn-glycero-3-phosphoethanolamine), NBD-PC (1-palmitoyl-2-{6-[(7-nitro-2-1,3-benzoxadiazol-4-yl)amino]hexanoyl}-sn-glycero-3-phosphocholine)), or a corresponding volume of solvent-only control (0.5% v/v ethanol) was added to triplicate wells of treated/untreated RBCs and incubated at room temperature in the dark, to allow NBD-lipids to translocate to the inner membrane leaflet. After 20 minutes, cells were washed twice with ice-cold 4% w/v lipid-free Bovine Serum Albumin (Sigma), to extract NBD-lipids remaining in the outer membrane leaflet. Cells were resuspended in PBS-G with 5 μg/mL Hoechst 33342 and incubated for 20 minutes on ice. Images and flow cytometry data were collected and processed as described above. The ATP-dependent fraction of lipid internalisation was calculated from the difference between total (untreated) and ATP-independent (vanadate-treated) lipid internalisation. Data were normalised between experiments (untreated uRBC set to 1) and analysed as specified. For figures comparing mean fluorescence intensity without normalisation (**Figs 1D** and **S1B**), data were fitted with a Linear Fixed Effects Model to calculate the Least Squared Means in RStudio, with date of experiment as a blocking factor to minimise the effect of fluctuations in machine settings [103]. Significance tests were computed by the ‘Difference of Least Squared Means’ function on this model.

The lipid internalisation assay was repeated on parasites isolated from their host RBCs by treatment with 0.15% w/v saponin (Sigma), after magnetic enrichment of iRBCs using a SuperMACS II Magnet (Miltenyi Biotec). The experiment was performed in malaria saline (135 mM NaCl, 5 mM KCl, 1 mM MgCl_2_, 20 mM glucose, 25 mM HEPES, pH. 7.1) instead of PBS-G. Images and flow cytometry data were collected and processed as described above. Data were normalised to the highest fluorescence value (untreated NBD-PC internalisation), and analysed in Prism 8 as specified.

### Annexin V staining

A culture containing uRBCs and 5–10% late-stage (trophozoite/schizont) iRBCs was washed twice in warm Ringer Solution (125 mM NaCl, 5 mM KCl, 1 mM MgSO_4_, 32 mM HEPES, 5 mM glucose, pH 7.4). RBCs were resuspended in Ringer Solution with 2.5 mM CaCl_2_, and incubated for 5 minutes with 0.5 mM sodium orthovanadate, 2 μM A23187 (Calbiochem), or the corresponding volume of buffer only. 5% v/v FITC-Annexin V stain was added to triplicate wells of each treatment, and incubated for 20 minutes at 37°C. Samples were resuspended in Ringer Solution (2.5mM CaCl_2_) with 5 μg/mL Hoechst 33342 and incubated for 20 minutes at 37°C. Images and flow cytometry data were collected and processed as described above. The percentage of uRBCs (Hoechst-negative) and iRBCs (Hoechst-positive) populations with FITC-Annexin V binding was calculated in FlowJo by setting a cut-off above unstained controls (**S3A Fig**). Data were analysed as specified.

### Calcium dye

All experiments were performed in glucose-containing media (≥5 mM), as it has been previously reported that glucose starvation causes Ca^2+^ influx in RBCs through a mechanism that is not otherwise active *in vitro* [87].

A culture containing uRBCs and 5–10% late-stage (trophozoite/schizont) iRBCs was washed in RPMI containing no sodium bicarbonate or Phenol Red (modified RPMI). Cells were incubated with 0.25 μM Cal520-AM dye (Abcam) or solvent-only control (0.06% v/v DMSO) in the modified RPMI for 60 minutes at 37°C on a rocker. Optimal dye concentration was determined in initial titration experiments. Cells were washed twice in modified RPMI and resuspended in 5 μg/mL Hoechst 33342 in modified RPMI, and incubated for 20 minutes at 37°C. Flow cytometry data were collected and processed as above. Data were normalised between experiments (uRBCs set to 1) and analysed using the Mann-Whitney Test.

For live cell imaging, RBCs were attached to glass-bottom culture dishes coated in 0.5 mg/mL Concanavalin A (Sigma), covered in warm modified RPMI, and kept at 37°C throughout imaging with a Deltavision Deconvolution microscope. Images were processed as specified.

For experiments analysing the effect of varying the concentration of calcium in the extracellular environment, cells were washed twice in warm Ringer Solution without Ca^2+^ (as for Annexin V staining). Cells were resuspended in Ringer Solution with a range of CaCl_2_ concentrations (0 mM, 0.42 mM, 1 mM, 2.5 mM, 5 mM), and incubated for 5 minutes with 0.5 mM sodium orthovanadate, 2μM A23187, or the corresponding volume of buffer only. 0.25 μM Cal520-AM dye or solvent-only control (0.06% v/v DMSO) was added to triplicate wells of each treatment, and incubated for 60 minutes at 37°C. Cells were washed twice in Ringer Solution (with corresponding CaCl_2_ concentration), resuspended in the same buffer with 5 μg/mL Hoechst 33342 and measured as above. Data were normalised between experiments (untreated uRBCs in 0 mM CaCl_2_ set to 1) and analysed as specified.

### Cholesterol staining

A culture containing uRBCs and 5–10% late-stage (trophozoite/schizont) iRBCs was washed twice in RPMI and three times in PBS-G. RBCs were incubated in 3% BSA (in PBS-G) for 45 minutes with a 1/40 dilution of 1 mg/mL recombinantly synthesised cholesterol-binding probe (θ-D4) conjugated to the fluorophore mCherry [76], a gift from Donatienne Tyteca, or with buffer alone. RBCs were washed three times in PBS-G, and incubated with 5 μg/mL Hoechst 33342 for 20 minutes. Events were measured on a LSRFortessa Flow Cytometer. mCherry fluorescence was detected at 561 nm ex/ 610 nm em. Hoechst fluorescence was detected at 350 nm ex/ 530 nm em), and data were processed as described above. Data were normalised between experiments (uRBCs set to 100%) and analysed using the Mann-Whitney Test. Images were collected at 37°C on a Deltavision Deconvolution microscope, and processed as specified.

### Monocyte uptake assay

Phagocytosis of RBCs was measured using the methods of [104] with modifications. iRBCs were separated using a SuperMACS II Magnet, giving populations >95% purity. iRBCs and uRBCs (kept under identical culture conditions without parasites) were stained with 50 μg/mL FITC (Sigma) in 0.5 M sodium bicarbonate (pH 9) for 15 minutes at room temperature, and washed three times in RPMI. RBCs were incubated for 20 minutes with 0.5 mM sodium orthovanadate or the corresponding volume of buffer only, and washed 3 times in culture media. Some RBCs were preincubated with 5 μg/mL purified Annexin V (BioLegend) for five minutes. Primary monocytes were purified from the buffy coat of whole blood on the day of collection using density gradient centrifugation and magnetically-tagged anti-CD14 beads (Miltenyi Biotec) with a SuperMACS II Magnet. Monocytes were tagged with anti-CD14 conjugated to PerCP (Miltenyi Biotec). RBCs and monocytes were combined in a 20:1 ratio and incubated for 30 minutes at 37°C, or 4°C for a no-phagocytosis control. Unphagocytosed RBCs were lysed in cold ammonium chloride (15 mM NH_4_Cl, 10 mM NaHCO_3_, 1 mM EDTA) for 3 minutes, and then fixed with 4% paraformaldehyde for 15 minutes. Samples were stained with 5 μg/mL Hoechst 33342, incubated for 20 minutes, and flow cytometry data was collected and processed as described above. The percentage of monocytes with at least one phagocytosed RBC was calculated based on FITC fluorescence, and normalised to the no phagocytosis control (incubated at 4°C). Data were analysed as specified. Images were collected and processed as described above.

## Supporting information

S1 Fig NBD-lipid internalisation at the RBC membrane differs between uRBCs and iRBCs. (**A**) Chemical structures of NBD-PS (top), NBD-PE (middle), and NBD-PC (bottom) used in this study. (**B**) NBD-lipid internalisation, measured by increase in NBD mean fluorescence intensity (MFI) of whole cells in flow cytometry after extraction of lipids remaining in the outer layer. Data are from Fig 1B, but shown without fold-change normalisation; units are arbitrary. Cells were treated with 0.5 mM vanadate in calcium-free media to measure only the ATP-independent portion of internalisation. Shown are Least Square Means (± 95% Confidence Interval). *n* = 3 independent experiments. (**C**) Example population histograms of flow cytometry data, showing internalisation of NBD-PS (left), NBD-PE (centre), and NBD-PC (right) in untreated RBCs (top row), and RBCs treated with 0.5 mM vanadate (bottom row). The black line indicates an arbitrary cut-off point, encompassing 98% of the uRBC population, and percentages represent the proportion of the iRBC population which falls above this cut-off point.
(TIF)

S2 Fig Z-sections of images from Fig 1A, showing subcellular localisation of NBD-lipids after internalisation, visualised by deconvolution fluorescence microscopy. NBD-lipid fluorescence was detected at 475 nm (ex)/ 525 nm (em) and Hoechst fluorescence (parasite DNA) was detected at 390 nm (ex)/ 435 nm (em). Scale bar = 4 μm. Z-sections taken 0.2 μm apart.
(TIF)

S3 Fig PS exposure and calcium dye fluorescence intensity differs between uRBCs and iRBCs, and changes with treatment of the calcium ionophore A23187. (**A**) Example population histograms of FITC-Annexin V fluorescence intensity by flow cytometry from Fig 1B, showing percentages of the population that fall above the cut-off point of background levels (no Annexin V control) indicated by the black line, for untreated (left), 0.5 mM vanadate (centre), and 2 μM A23187 (right). (**B**) Mean percentage (± S.D.) of RBCs exposing PS in the membrane outer leaflet, measured by Annexin V staining above background levels (no Annexin V control), with and without 2 μM A23187. * = p < 0.05; ** = p < 0.01 (ANOVA). *n* = 3 independent experiments. (**C**) Calcium dye fluorescence intensity in RBCs treated with 2 μM A23187 across different extracellular Ca^2+^ concentrations. Shown are mean values (± S.D.), NS = not significant; * = p < 0.05; ** = p < 0.01; *** = p < 0.001 (ANOVA). *n* = 3 independent experiments.
(TIF)

S4 Fig Representative monocytes from Fig 5A, with phagocytosed iRBCs from 3D7 wildtype, CS2 wildtype, and CS2ΔSBP1 parasite strains. FITC fluorescence was detected at 475 nm (ex)/ 525 nm (em), PerCP fluorescence (anti-CD14) was detected at 475 nm (ex)/ 679 nm (em), and Hoechst fluorescence (DNA) was detected at 390 nm (ex)/ 435 nm (em). Scale bar = 6 μm.
(TIF)

S5 Fig Z-sections of images from Fig 6B, showing subcellular localisation of NBD-lipids after internalisation, visualised by deconvolution fluorescence microscopy. NBD-lipid fluorescence was detected at 475 nm (ex)/ 525 nm (em) and Hoechst fluorescence (parasite DNA) was detected at 390 nm (ex)/ 435 nm (em). Scale bar = 4 μm. Z-sections taken 0.2 μm apart.
(TIF)

S1 Data Numerical data for figures. (XLSX)
