# Supplementary figures and images for: Breakdown in membrane asymmetry regulation leads to monocyte recognition of P. falciparum-infected red blood cells
Source: PLoS Pathog. 2021 Feb 18;17(2):e1009259. doi: 10.1371/journal.ppat.1009259 (PMC7891792; doi:10.1371/journal.ppat.1009259)

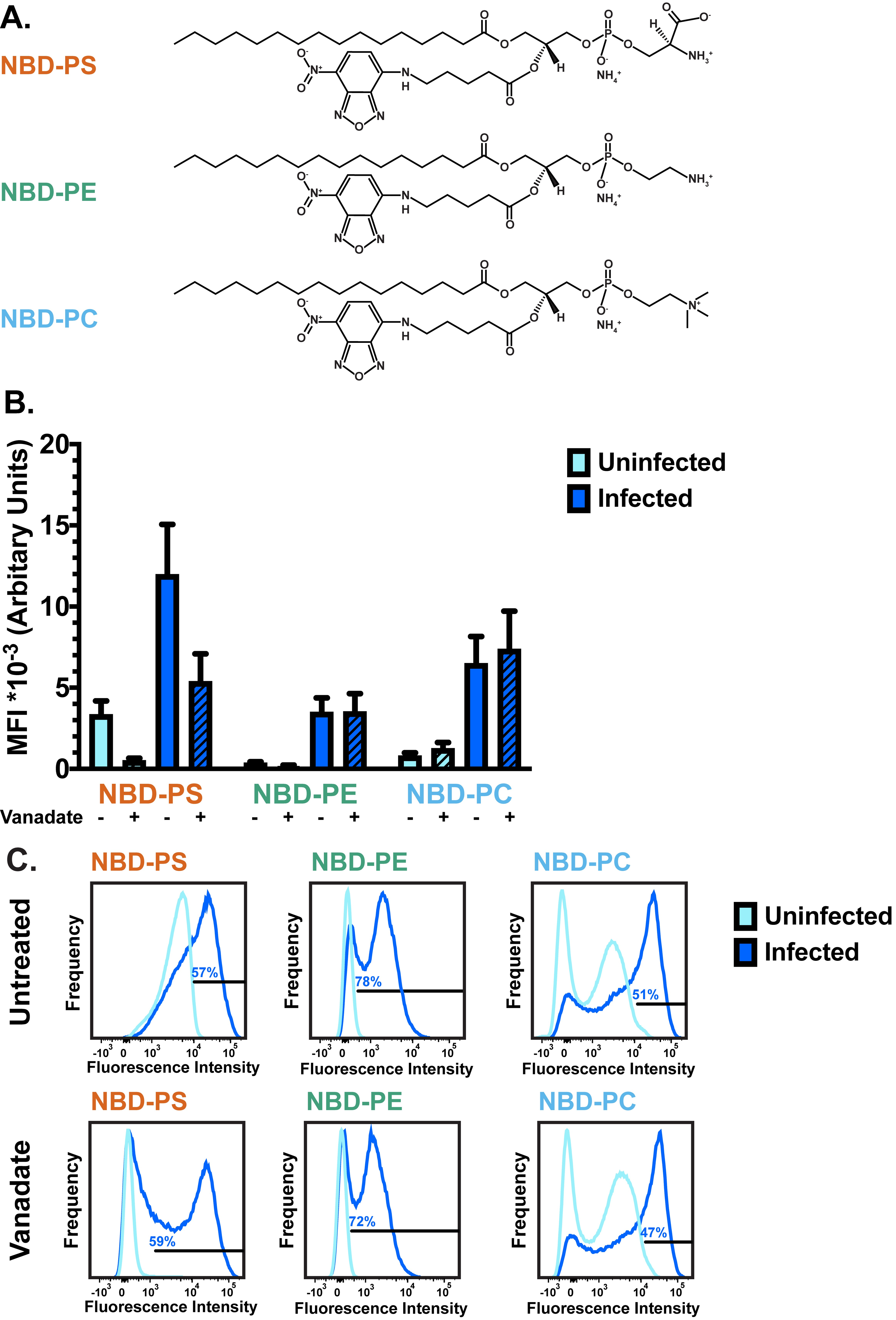

Supplement: S1 Fig — (A) Chemical structures of NBD-PS (top), NBD-PE (middle), and NBD-PC (bottom) used in this study. (B) NBD-lipid internalisation, measured by increase in NBD mean fluorescence intensity (MFI) of whole cells in flow cytometry after extraction of lipids remaining in the outer layer. Data are from Fig 1B, but shown without fold-change normalisation; units are arbitrary. Cells were treated with 0.5 mM vanadate in calcium-free media to measure only the ATP-independent portion of internalisation. Shown are Least Square Means (± 95% Confidence Interval). n = 3 independent experiments. (C) Example population histograms of flow cytometry data, showing internalisation of NBD-PS (left), NBD-PE (centre), and NBD-PC (right) in untreated RBCs (top row), and RBCs treated with 0.5 mM vanadate (bottom row). The black line indicates an arbitrary cut-off point, encompassing 98% of the uRBC population, and percentages represent the proportion of the iRBC population which falls above this cut-off point. (TIF) [file ppat.1009259.s001.tif]

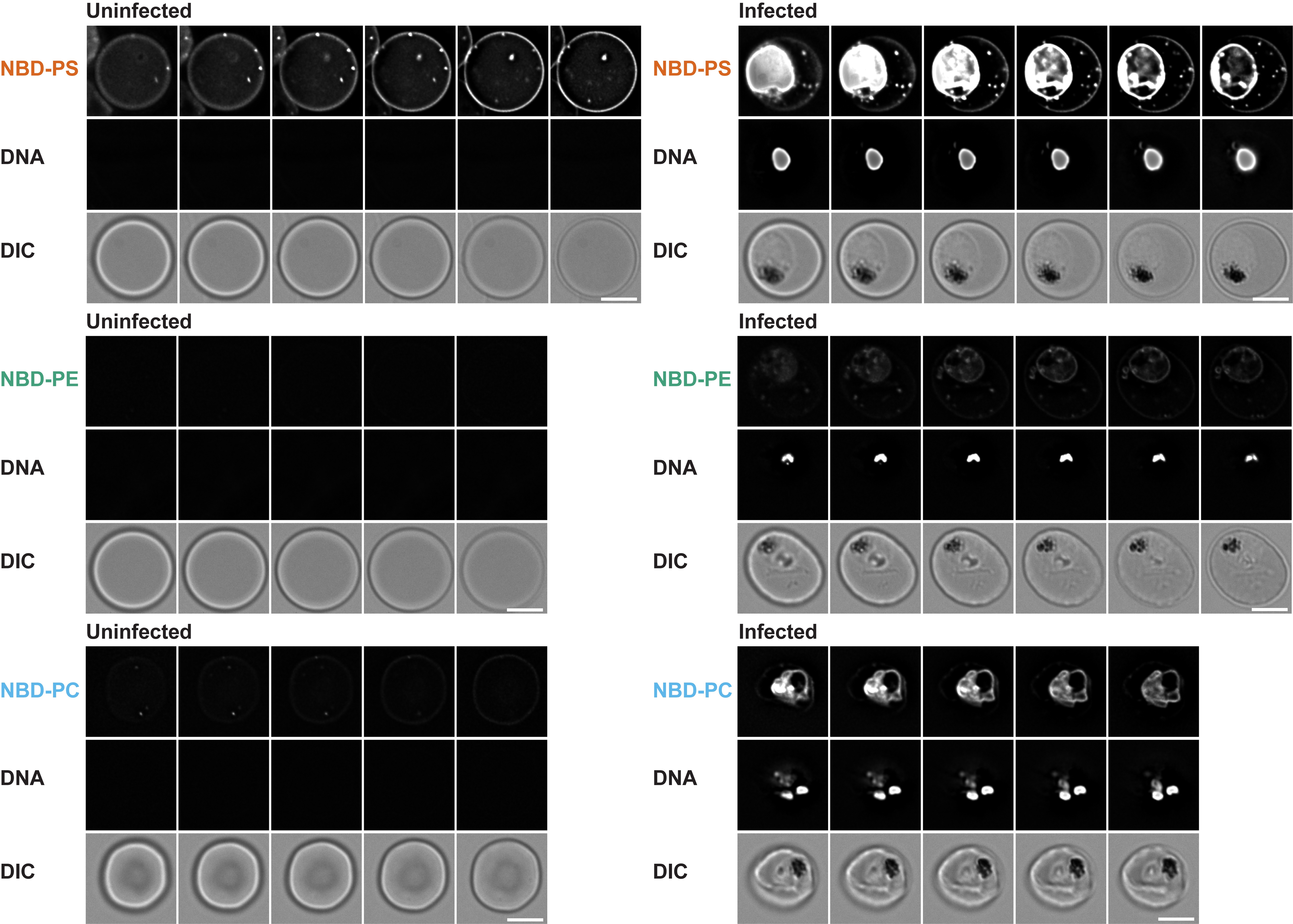

Supplement: S2 Fig — NBD-lipid fluorescence was detected at 475 nm (ex)/ 525 nm (em) and Hoechst fluorescence (parasite DNA) was detected at 390 nm (ex)/ 435 nm (em). Scale bar = 4 μm. Z-sections taken 0.2 μm apart. (TIF) [file ppat.1009259.s002.tif]

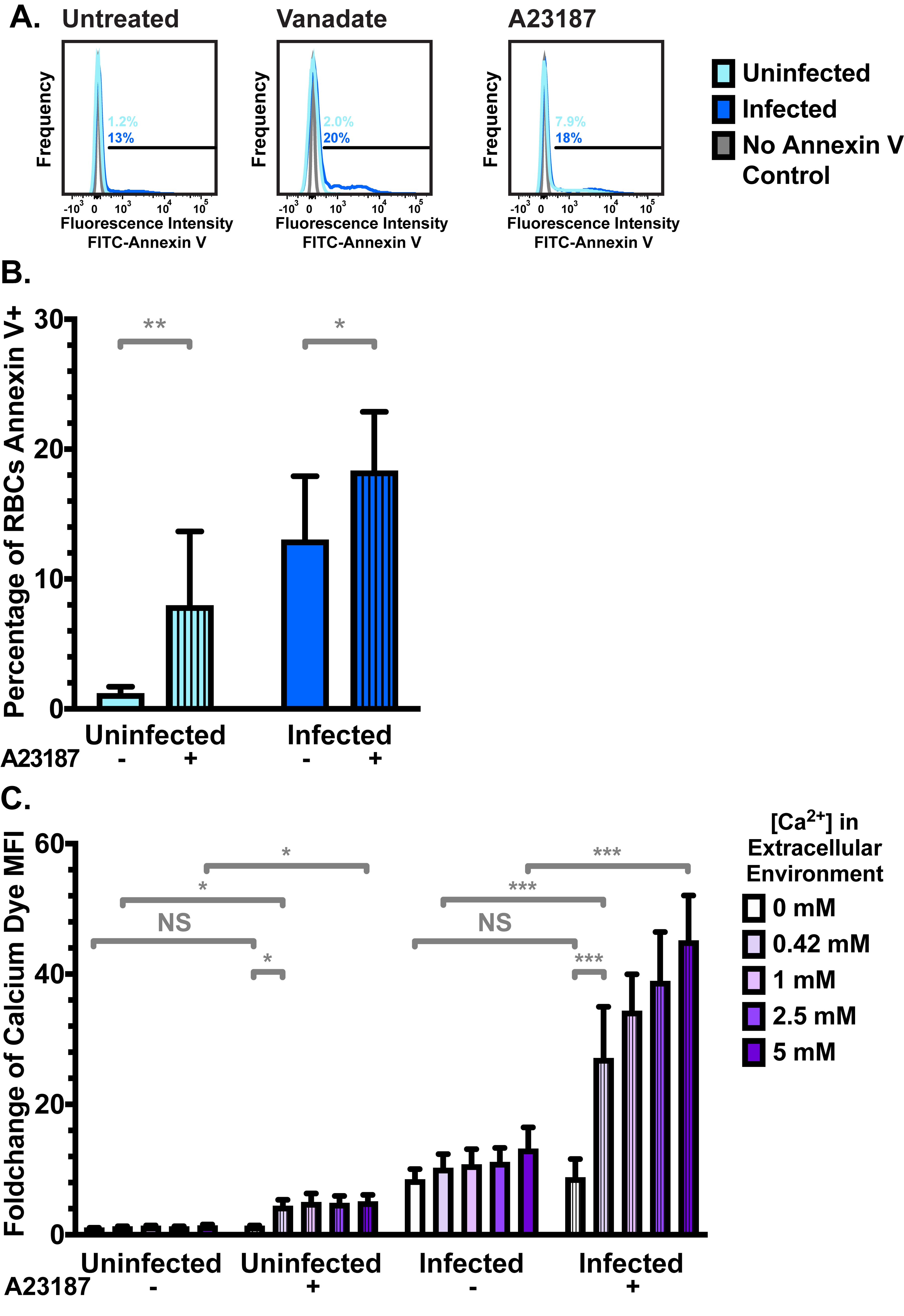

Supplement: S3 Fig — (A) Example population histograms of FITC-Annexin V fluorescence intensity by flow cytometry from Fig 1B, showing percentages of the population that fall above the cut-off point of background levels (no Annexin V control) indicated by the black line, for untreated (left), 0.5 mM vanadate (centre), and 2 μM A23187 (right). (B) Mean percentage (± S.D.) of RBCs exposing PS in the membrane outer leaflet, measured by Annexin V staining above background levels (no Annexin V control), with and without 2 μM A23187. * = p < 0.05; ** = p < 0.01 (ANOVA). n = 3 independent experiments. (C) Calcium dye fluorescence intensity in RBCs treated with 2 μM A23187 across different extracellular Ca2+ concentrations. Shown are mean values (± S.D.), NS = not significant; * = p < 0.05; ** = p < 0.01; *** = p < 0.001 (ANOVA). n = 3 independent experiments. (TIF) [file ppat.1009259.s003.tif]

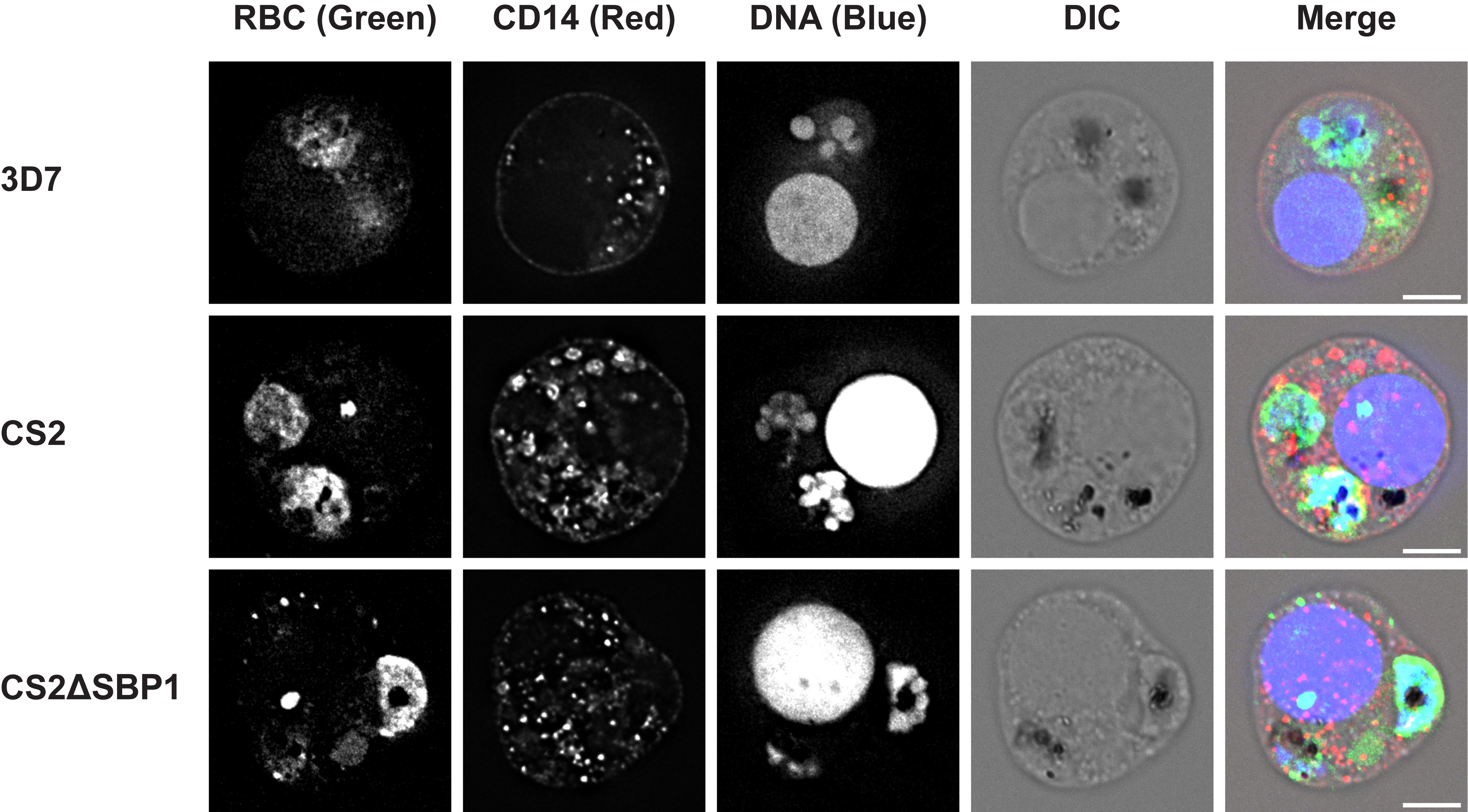

Supplement: S4 Fig — FITC fluorescence was detected at 475 nm (ex)/ 525 nm (em), PerCP fluorescence (anti-CD14) was detected at 475 nm (ex)/ 679 nm (em), and Hoechst fluorescence (DNA) was detected at 390 nm (ex)/ 435 nm (em). Scale bar = 6 μm. (TIF) [file ppat.1009259.s004.tif]

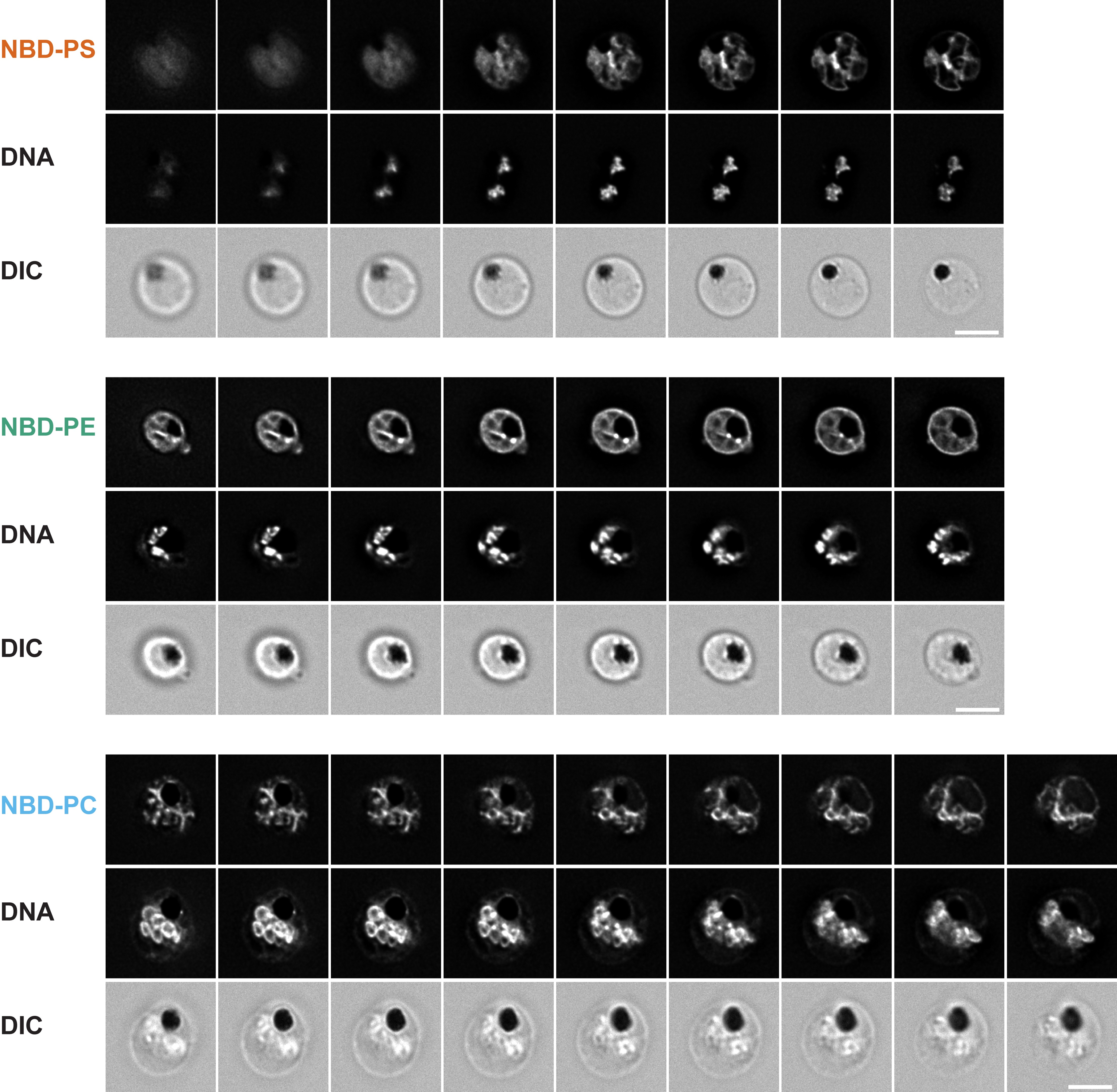

Supplement: S5 Fig — NBD-lipid fluorescence was detected at 475 nm (ex)/ 525 nm (em) and Hoechst fluorescence (parasite DNA) was detected at 390 nm (ex)/ 435 nm (em). Scale bar = 4 μm. Z-sections taken 0.2 μm apart. (TIF) [file ppat.1009259.s005.tif]
